# Dynamic EMT: a multi‐tool for tumor progression

**DOI:** 10.15252/embj.2021108647

**Published:** 2021-08-30

**Authors:** Simone Brabletz, Harald Schuhwerk, Thomas Brabletz, Marc P. Stemmler

**Affiliations:** ^1^ Department of Experimental Medicine 1 Nikolaus‐Fiebiger Center for Molecular Medicine Friedrich‐Alexander University of Erlangen‐Nürnberg Erlangen Germany

**Keywords:** EMT, MET, ZEB1, ZEB2, SNAIL, SLUG, TWIST, cancer, invasion, metastasis, signaling pathways, cell plasticity, tumor stemness, hybrid EMT, partial EMT, Cancer, Cell Adhesion, Polarity & Cytoskeleton, Chromatin, Epigenetics, Genomics & Functional Genomics

## Abstract

The process of epithelial–mesenchymal transition (EMT) is fundamental for embryonic morphogenesis. Cells undergoing it lose epithelial characteristics and integrity, acquire mesenchymal features, and become motile. In cancer, this program is hijacked to confer essential changes in morphology and motility that fuel invasion. In addition, EMT is increasingly understood to orchestrate a large variety of complementary cancer features, such as tumor cell stemness, tumorigenicity, resistance to therapy and adaptation to changes in the microenvironment. In this review, we summarize recent findings related to these various classical and non‐classical functions, and introduce EMT as a true tumorigenic multi‐tool, involved in many aspects of cancer. We suggest that therapeutic targeting of the EMT process will—if acknowledging these complexities—be a possibility to concurrently interfere with tumor progression on many levels.

## Graphical abstract

This review contrasts classical and non‐classical roles of epithelial‐mesenchymal transition in diverse aspects of cancer cell biology.

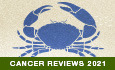

## Introduction

**Figure 1:**
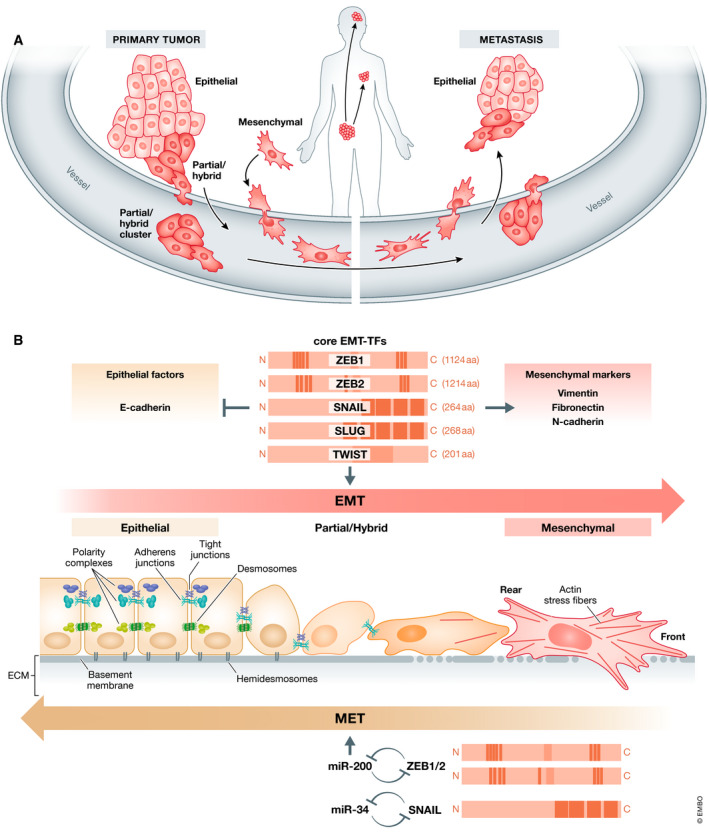
Classical EMT functions and cancer (A) EMT frequently occurs at the invasive front of epithelial tumors, destroys the well‐defined epithelial structures, and allows the cancer cells to migrate, invade the tissue, and intravasate in blood or lymphatic vessels. Tumor cells on their way through the body can travel as mesenchymal single cells, as cell clusters exhibiting partial EMT or as more epithelial cell clusters headed by a mesenchymal leader cell. At the secondary site, the cells extravasate and colonize the distant organ, where MET allows the outgrowth to macrometastases. (B) EMT is induced mainly by a set of transcription factors (EMT‐TFs) like ZEB1, ZEB2, SNAIL, SLUG and TWIST that differ in protein structure, size, and individual functions. All of them are repressors of epithelial factors like E‐cadherin and activate mesenchymal markers like Vimentin, Fibronectin or N‐cadherin. Epithelial cells displaying apical–basal polarity are held together by tight junctions, adherens junctions, and desmosomes and are anchored to the underlying basement membrane by hemidesmosomes. They express three different polarity complexes that together with the junctional molecules maintain epithelial cell polarity. In the classical EMT, expression of EMT‐TFs leads to inhibition of major components of these epithelial structures and concomitantly activates the expression of genes associated with the mesenchymal state. Cells gain front–rear polarity, display actin stress fibers, become motile and acquire invasive capacities. Notably, tumor cells very rarely switch to a completely mesenchymal phenotype, but fluently convert between various intermediate states displaying certain mesenchymal features but keeping partial sets of epithelial characteristics. Further, EMT is a reversible process. Mesenchymal cells can revert to the epithelial state undergoing MET. An important role in the execution of MET is played by microRNAs of the miR‐200 and mir‐34 families that are regulated in double‐negative feedback loops with the EMT‐TFs ZEB1/2 and SNAIL, respectively, that serve to reinforce either the epithelial or the mesenchymal state.

Epithelial‐to‐mesenchymal transition (EMT) describes the transdifferentiation of stationary epithelial cells to a mesenchymal, motile phenotype and was initially observed in early development (Hay, 1995). Here, EMT contributes to embryonal processes like gastrulation, neural crest formation, or heart development (Thiery *et al*, 2009; Nieto *et al*, 2016). The program is also crucial for physiological processes like wound healing (Arnoux *et al*, 2008) and tissue homeostasis (Ahmed *et al*, 2006). Importantly, pathological reactivation of the EMT process plays a fundamental role in diseases like organ fibrosis or cancer progression to metastasis (Fig 1A), which is the focus of this review.

**Figure 2:**
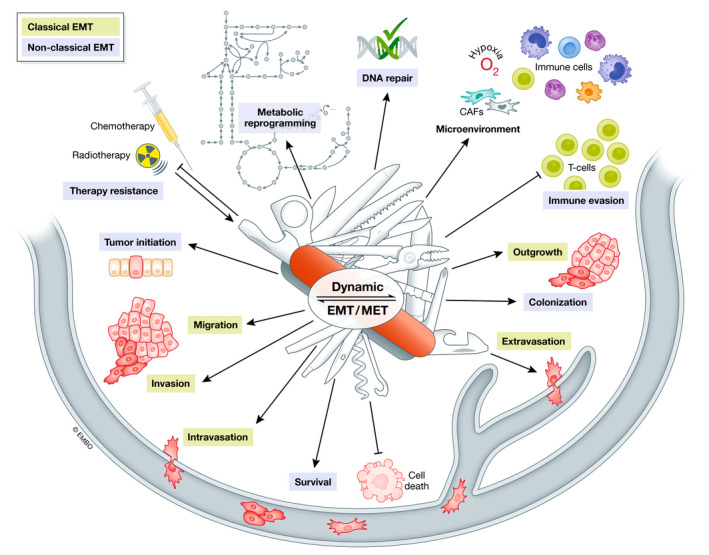
Dynamic EMT as a multi‐tool for tumor progression Overview summarizing the multiple oncogenic EMT functions in the course of tumor progression. The classical EMT functions allow cancer cell to migrate, invade, intra‐ and extravasate blood and lymphatic vessels. At the distant sites, MET enables the outgrowth of macrometastases. The non‐classical EMT traits support tumor initiation as well as metastatic colonization. Throughout the whole process of tumor progression, they help the cells to cope with changing conditions by metabolic reprogramming, enhanced survival via altered DNA repair and prevention of cell death, immune evasion and improved resistance to chemo‐ and radiotherapy. Importantly, EMT is not only supporting the cancer cells to handle changing environmental conditions, but is also induced by extracellular signals from, e.g., CAFs or immune cells in the microenvironment or by therapeutic approaches.

Cancer is an extremely complex and diverse disease not only varying between entities, but also within the same entity, between different subtypes, and even within subtypes. Notably, tumors display not only spatial but also temporal heterogeneity within the same individual that can be elicited, e.g., via the occurrence of consecutive mutations and clonal evolution (McGranahan & Swanton, 2017). However, cancer cell plasticity, allowing continuous and reversible adaption to ever‐changing conditions, is mediated by the EMT process that is not genetically fixed, depending on accumulating mutations, but is epigenetically orchestrated by signals from the microenvironment, rendering the whole program reversible (by activating mesenchymal–epithelial transition; MET) and highly dynamic (see overview in Fig 2).

The EMT program is mainly executed by a core set of EMT‐activating transcription factors (EMT‐TFs) including SNAIL (also SNAI1) and SLUG (also SNAI2), the basic helix–loop–helix factors TWIST1 (TWIST) and TWIST2 and the zinc finger E‐Box binding homeobox factors ZEB1 and ZEB2. All these factors share the ability to repress epithelial genes like the E‐cadherin encoding gene *CDH1* via binding to E‐Box motifs in their cognate promoter regions (Nieto *et al*, 2016) as shown for SNAIL (Batlle *et al*, 2000; Cano *et al*, 2000), TWIST (Yang *et al*, 2004), ZEB1 (Eger *et al*, 2005), and ZEB2 (Comijn *et al*, 2001). In parallel, the EMT‐TFs directly or indirectly activate genes associated with a mesenchymal phenotype, including *VIM* (Vimentin), *FN1* (Fibronectin), and *CDH2* (N‐cadherin) (Nieto *et al*, 2016; Dongre & Weinberg, 2019) (Fig 1B). However, many functions are not shared, but executed by distinct EMT‐TFs, as they differ in aspects like expression patterns or extremely different protein size and structure (Stemmler *et al*, 2019).

Beyond the “classical” EMT traits, the gain of motility, and invasive capacities, the widespread importance of EMT‐TFs in cancer biology is indicated by additional pleiotropic functions (Brabletz *et al*, 2018). EMT‐TFs have been shown to maintain stemness properties and to increase tumorigenicity, linking them to the concept of cancer stem cells (CSCs). Additionally, EMT‐TFs are involved in DNA repair, the escape from senescence and apoptosis, therapy resistance, and immune evasion resulting in a pro‐survival phenotype, providing an advantage under various types of stress conditions.

Altogether, the classical EMT functions together with the very diverse non‐redundant context‐dependent non‐classical functions of EMT‐TFs that are dynamically regulated also by the tumor microenvironment (TME) allow the cancer cells to permanently adapt to changing conditions (Puisieux *et al*, 2014). Consequently, therapeutic intervention with EMT/plasticity will provide the opportunity to fight many aspects of tumor progression at a single blow.

In this review, we summarize all these different EMT functions in cancer biology and highlight the resulting clinical implications.

## Classical/core EMT functions

### Migration and invasion

In a normal epithelial tissue, the cells form continuous protective sheets that are crucial for their structural integrity. The connection between epithelial cells is mediated by different cell junction complexes including adherens junction, desmosomes, and tight junctions that seal the cells, are located apical to the adherens junctions to support epithelial polarity and constitute a barrier for solutes and water. This apical‐basal polarity is fundamental for tissue function, and has been defined as “asymmetry” within cells and epithelial tissues. Polarity complexes, including the Par, the Crumbs, and the Scribble complexes, ensure the proper organization of apical versus basolateral domains in the epithelial cell (Huang *et al*, 2012). Of note, some of the genes that control epithelial cell polarity also regulate spindle orientation and division mode in stem cells (Martin‐Belmonte & Perez‐Moreno, 2011) (Fig 1B).

Elicited by the TME, the activation of EMT is fundamental for the transition toward malignancy, accompanied by substantial cellular changes on multiple levels. Cell–cell contacts become deconstructed through the repression of *CDH1*, encoding epithelial cadherin (E‐cadherin), the main constituent of adherens junctions, and of genes coding for other epithelial junction molecules.

As a consequence of the disintegration of all epithelial junction complexes and of direct transcriptional repression of several members of the Crumbs and the Scribble complexes, apical–basal polarity is lost (Aigner *et al*, 2007; Moreno‐Bueno *et al*, 2008; Spaderna *et al*, 2008; Lamouille *et al*, 2014) (Fig 1B). This coincides with profound cytoskeletal reorganization like apical constriction, the formation of actin stress fibers, and the conversion of cell morphology from cuboidal or columnar shapes to more spindle‐like elongated forms with front–rear polarity, resulting in a gain of motility for the tumor cells (Moreno‐Bueno *et al*, 2008). Newly formed actin‐rich membrane protrusions like lamellipodia and filopodia support cell movement. To invade the surrounding tissues, e.g., TWIST or ZEB1 also induce the formation of invadopodia, specialized filopodia with proteolytic function (Yilmaz & Christofori, 2009; Eckert *et al*, 2011; Ridley, 2011; Sundararajan *et al*, 2015). This is supported by induction of matrix metalloproteases by EMT‐TFs, essential for degradation of the basement membrane and the extracellular matrix of adjacent tissues (Miyoshi *et al*, 2004; Miyoshi *et al*, 2005; Huang *et al*, 2009) (Fig 1B). Additionally, EMT inducers prevent basement membrane synthesis by directly repressing the transcription of its components (Spaderna *et al*, 2006). All these classical EMT events cause the loss of epithelial integrity of the tissue, allow invasion and dissemination of cancer cells, and thus execute the first step of the metastatic cascade (Fig 1A).

### MET: metastatic colonization and outgrowth

In 1990, Fearon and Vogelstein proposed a meanwhile classical genetic model for colorectal tumorigenesis. They described tumor development to be the continuous deterioration of initially normal epithelial cells toward greater malignancy driven by the stepwise accumulation of mutations and hypothesized that this process perpetuates during the last step of tumor progression from an established malignant carcinoma to distant metastases, implying that distant metastases are the most degenerated tissues of the malignancy (Fearon & Vogelstein, 1990). However, all efforts to identify specific metastasis‐associated mutations remained unsuccessful.

**Figure 3:**
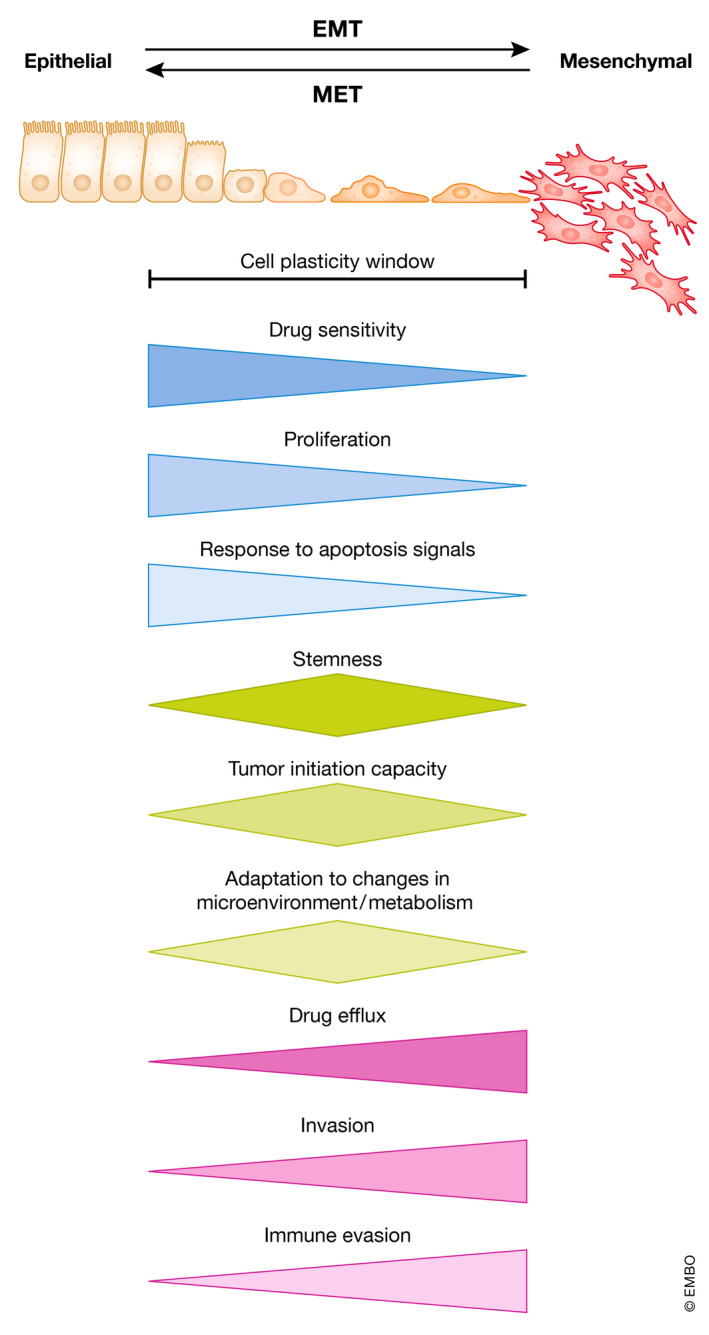
Cellular plasticity of tumor cells is governed by EMT and provided in a window of partial EMT Dynamic induction of EMT and MET changes cellular phenotypes of carcinoma cells. Drug sensitivity, proliferation, and response to apoptosis signals are highest in more epithelial states, whereas drug efflux, invasion, and immune evasion are highest in more mesenchymal states. A hybrid EMT state provides maximal stemness, tumor initiation capacity, and ability to adapt to environmental changes. Note that in extreme epithelial and mesenchymal states, features like stemness, tumor initiation, and colonization are lost.

Rather, already twenty years ago, it was observed that metastases, compared with the de‐differentiated nature of the invasion front of the primary tumor, exhibit a re‐differentiated epithelial morphology, similar to the center of the primary tumor (Brabletz *et al*, 2001). These findings led to the hypothesis that de‐differentiation and EMT during invasion is a transient condition and that an opposing process of epithelial re‐differentiation or MET needs to be initiated and is advantageous for the formation of distant macrometastases (Figs 1A and 2). But why do metastases re‐differentiate? Invasive, de‐differentiated cancer cells were shown to be growth arrested, whereas proliferation was detected in re‐differentiated metastasis, suggesting that EMT must be reversed in order to allow colonization and growth (Brabletz *et al*, 2001). This is supported by the fact that EMT‐TFs can directly inhibit proliferation (Thiery *et al*, 2009) (Fig 3). There are also several publications confirming the relevance of MET for metastatic outgrowth (Chaffer *et al*, 2006; Korpal *et al*, 2011; Ocana *et al*, 2012; Tsai *et al*, 2012). Importantly, the occurrence of MET is in perfect accordance with the failure to identify EMT‐causing mutations, but is attributed to the transcriptional and epigenetic regulation of the process.

Whereas EMT induction has been investigated in great detail (see below), less is known about the trigger of the reverse MET process. Is it just lack of EMT‐inducing stimuli coupled to reduced expression of EMT‐TFs? Several studies could show that knockdown of the EMT‐TF *ZEB1* is sufficient to elicit MET *in vitro* in carcinoma cell lines of different entities (Eger *et al*, 2005; Spaderna *et al*, 2006; Spaderna *et al*, 2008) and that depletion of *Zeb1* in a genetic mouse model of pancreatic cancer fixes the tumor cells in an epithelial MET state (Krebs *et al*, 2017). Once expression of ZEB‐family members (*ZEB1* and *ZEB2*) declines, their reduction is reinforced by a double‐negative feedback loop with the miR‐200 family of microRNAs. During EMT, ZEB factors transcriptionally repress the genes of all miR‐200 family members. *Vice versa*, miR‐200 family members inhibit expression of ZEB1/2 at the post‐transcriptional level (Fig 1B). Thus, the bidirectional transitions between EMT and MET states are potentiated by the ZEB/miR‐200 circuit (Bracken *et al*, 2008; Burk *et al*, 2008; Wellner *et al*, 2009). This branch of MET was shown to be inducible via the tumor‐suppressor p53, that directly activates miR‐200s transcription (Kim *et al*, 2011). Upregulation of miR‐200 family members has different consequences. It exerts both an invasion‐ and migration‐inhibiting, thus tumor‐suppressing function (Peter, 2009), and promotes metastatic colonization (Korpal *et al*, 2011). Importantly, these findings are not controversial, but highlight the dynamic nature of the EMT‐MET process adapting the tumor cells to the respective conditions. Similar to the ZEB/miR‐200 negative feedback loop, SNAIL and the miR‐34 family constitute additional epithelial plasticity regulatory loops (Siemens *et al*, 2011; Diaz‐Lopez *et al*, 2014) (Fig 1B). Of note, the described microRNAs target not only EMT‐TFs themselves, but also genes like *BMI1*, *CD44*, *CD133*, *JAG1*, or *MYC* that are involved in tumor‐relevant “non‐classical” EMT functions like stemness and survival as discussed below (Brabletz & Brabletz, 2010; Brabletz *et al*, 2011).

Since the metastatic cascade is an extremely complex and slow multistep process that can take many years and requires very different, sometimes apparently opposing traits from the tumor cells in a spatiotemporal manner, valid investigation remains challenging and must rely on mouse tumor models. Recently, several studies were focusing on the spatiotemporal regulation of EMT/MET using elegant mouse models. In 2012, Tsai et al (2012) demonstrated that EMT induction, in this case via TWIST, supports tumor cell dissemination in a mouse model of skin cancer, but subsequent *Twist1* downregulation and MET was necessary for colonization and formation of macrometastases (Tsai *et al*, 2012) (Fig 3). Another study described the necessity of an EMT in the MMTV‐PyMT mouse model of breast cancer to disseminate to the lung. Here, the EMT‐state cancer cells at the metastatic niche activate local fibroblasts that in turn induce epithelial re‐differentiation (MET) for outgrowth of macrometastases (del Pozo Martin *et al*, 2015). Further, Esposito and colleagues found that E‐selectin mediated cell adhesion of bone vascular niche cells to cancer cells elicits MET and promotes bone metastasis (Esposito *et al*, 2019).

In summary, many reports support the occurrence and significance of MET in metastatic colonization and outgrowth (Fig 3). However, its regulation and detailed functions still need further investigation.

## Partial EMT

EMT and MET have long been viewed as a binary switch between separate epithelial or mesenchymal cell populations. In the past, this narrow perspective challenged the concept of EMT as a major means in metastasis. One example for this is the analysis of Fischer *et al* using *Fsp1*‐Cre driven lineage tracing in a genetic mouse model of breast cancer. Based on the finding that lung metastases mainly consist of tumor cells that had never switched to a full mesenchymal Fsp1+ phenotype, the authors concluded that EMT is not important for this process (Fischer *et al*, 2015).

However, nowadays it is accepted that, although reactivated in many cancer types, EMT is rarely fully executed in tumor cells, and end‐stage markers such as Vimentin are often not expressed. The process is rather gradual and often remains incomplete, termed partial or hybrid EMT (Nieto *et al*, 2016; Pastushenko & Blanpain, 2019; Yang *et al*, 2020) (Fig 1B). Over the last years, more and more studies report *in vivo* detection of cancer cells carrying a combination of both epithelial and mesenchymal markers. Already in the 1990s, early analyses reported observations of cancer cells exhibiting only partial loss of E‐cadherin during invasion (Mareel *et al*, 1992; Birchmeier & Behrens, 1994). Later, additional publications described circulating tumor cells (CTCs) with simultaneous expression of epithelial and mesenchymal markers (Yu *et al*, 2013). Similarly, partial EMT of cells within a tumor was identified by co‐expression of epithelial (EpCAM+) and mesenchymal (Vim+) marker genes in an autochthonous murine prostate cancer model (Ruscetti *et al*, 2015). Partial EMT was also linked to metastasis via single‐cell transcriptomics in head and neck cancer (Puram *et al*, 2017). Another group identified different tumor transition states occurring during EMT in cancer progression and introduced the term “hybrid” EMT (Pastushenko *et al*, 2018; Pastushenko & Blanpain, 2019; Pastushenko *et al*, 2021) (Fig 1B). Moreover, Bornes and colleagues demonstrated that *Fsp1‐*Cre mediated lineage tracing as used by Fischer *et al* (2015) is incapable in detecting the majority of disseminating cells that are in a partial/hybrid EMT state (Fischer *et al*, 2015; Bornes *et al*, 2019). Importantly, this lack of full EMT does not mean that the process is not important. There is direct *in vivo* evidence that, e.g., the lack of Z*eb1* expression in a genetic mouse model of pancreatic cancer that traps the cancer cells in an epithelial phenotype profoundly suppresses invasion and metastasis (Krebs *et al*, 2017). In mouse mammary carcinoma cells, SNAIL expression is crucial for *Zeb1* induction and metastatic dissemination (Ye *et al*, 2015). Similarly, in an oncogene‐induced mammary tumor model, Xu *et al* (2017) found TWIST to be required for the expression of other EMT‐TFs in a small subset of tumor cells to induce partial EMT and metastasis (Xu *et al*, 2017).

Interestingly, different modes of cancer cell invasion that involve EMT at various levels have been observed. Cancer cells can invade in groups forming rather epithelial clusters. Indeed, this type of “collective migration” might be even more common than single‐cell dissemination, as supported by several studies using lineage tracing approaches and detection of clustered tumor cells in circulation (Friedl *et al*, 2012; Aceto *et al*, 2014; Cheung *et al*, 2016). Nevertheless, despite the epithelial appearance, either characteristic of a partial EMT is also detectable in these migrating cell clusters (Aiello *et al*, 2018), or the epithelial clusters follow mesenchymal “leader” cells that pave the way for their more epithelial “follower” cells (Matise *et al*, 2012; Chen *et al*, 2020) (Fig 1A).

## Non‐classical EMT features

Besides the classical features to drive changes in cellular phenotypes, EMT is also involved in regulating various additional aspects of tumorigenesis (Fig 2).

### Regulation of stemness

Normal tissue homeostasis is dependent on the function of stem cells that serve as a source to replenish dying committed and terminally differentiated cells in a tissue. The observation that tumor heterogeneity is maintained after single tumor cell transplantations into mice, prompted to adapt normal stem cell biology also to tumors (Reya *et al*, 2001). Simplified, cancer cell stemness is measured by the capability of cell fractions to form tumors in mice. Strikingly, tumor initiation capacity is increasing with EMT activation by stem cell features provided by EMT‐TFs within the cell plasticity window (Puisieux *et al*, 2014; Nieto *et al*, 2016; Shibue & Weinberg, 2017; Wilson *et al*, 2020) (Fig 3). Overexpression of *SNAI1*, *TWIST1*, and *ZEB1* in breast cancer is sufficient to increase the CD44^+^/CD24^lo^ stem cell pool, resulting in increased sphere forming capacity *in vitro*, as well as elevated tumorigenicity and metastasis *in vivo* (Mani *et al*, 2008; Morel *et al*, 2008). Moreover, SLUG determines stem cell states in the healthy mammary gland and converts luminal cells to stem cells in both healthy and tumor cell settings (Guo *et al*, 2012). Similar results were obtained for pancreatic cancer, where ZEB1 expression is a key determinant of cancer cell stemness and the reciprocal ZEB1/miR‐200 feedback loop is controlling the expression of genes of stemness factors including *BMI1* and *SOX2* (Shimono *et al*, 2009; Wellner *et al*, 2009; Krebs *et al*, 2017). Similarly, in squamous cell carcinoma (SCC) TWIST is regulating *BMI1* to cooperatively repress *CDKN2A* (p16^INK4A^) and promote tumor initiation capacities (Yang *et al*, 2010). Recently, the protocadherin FAT1 was identified as one additional player in regulating EMT and stemness. *FAT1* is frequently inactivated resulting in increased tumor stemness and metastasis in a mouse model of SCC involving CAMK2, CD44, and SRC activities that induce nuclear translocation of YAP1 and ZEB1 (Pastushenko *et al*, 2021). Interestingly, another important EMT promoting transcription factor, PRRX1, promotes EMT during development and in breast cancer cells, but its loss is required for cancer cell metastasis, thereby uncoupling EMT/migration from stemness features (Ocana *et al*, 2012). However, PRRX1 isoform switching is the driving force of invasion and metastasis in pancreatic cancer (Takano *et al*, 2016). Mechanistically, defined cellular states within the EMT cell plasticity realm correlate with gradually increasing efficiencies in metastatic seeding and invasion (Pastushenko *et al*, 2018), in line with the idea of partial/hybrid EMT states.

In addition to the regulation of tumor stemness and colonization (Shibue & Weinberg, 2009; Shibue *et al*, 2013; Nieto *et al*, 2016; Stemmler *et al*, 2019; Wilson *et al*, 2020), EMT is involved in driving tumor initiation and malignant transformation. *TWIST1* and *ZEB1* upregulation in RAS transformed mammary and bronchial epithelial cells is sufficient to unleash malignancy favoring formation of aggressive undifferentiated tumors (Morel *et al*, 2012; Liu *et al*, 2014b; Larsen *et al*, 2016). Moreover, EMT can induce reduction in KRAS dependency of tumor cells (KRAS addiction) and different ZEB1 thresholds drive KRAS‐dependent tumor initiation and metastasis capacities (Singh *et al*, 2009; Liu *et al*, 2014b). The oncogenic effects of EMT‐TF activities are even more evident by ectopic activation of *Zeb2* in the intestinal epithelium of transgenic mice. Elevation of ZEB2 generates invasive colorectal cancer in absence of cooperating genetic defects (Slowicka *et al*, 2020).

### Therapy resistance

Loss of durable therapeutic efficacy and tumor relapse after initial successful treatment are major obstacles in the battle against cancer. Conventional therapy is favorably eliminating differentiated non‐stem cell‐like cells, but fails to deplete cancer cells with stem cell properties. EMT activation in these cells is controlling therapy resistance on multiple levels (Singh & Settleman, 2010; Shibue & Weinberg, 2017; Santamaria *et al*, 2019; Dudas *et al*, 2020). EMT gene signatures and acquisition of therapy resistance are strongly correlated, e.g., for standard and for targeted therapy with EGFR or PI3K inhibitors (Creighton *et al*, 2009; Farmer *et al*, 2009; Byers *et al*, 2013). For example, gemcitabine‐resistant Panc1 pancreatic cancer cells exhibit EMT characteristics and become sensitized upon *ZEB1* knockdown (Wellner *et al*, 2009). Mechanistically, common routes toward drug resistance include increasing drug efflux or evading apoptosis and anoikis (Fig 3). The former is mediated by activating the expression of members of the ATP‐binding cassette (ABC) transporter family, which are transcriptionally regulated by the EMT‐TFs TWIST, SNAIL, and FOXC1 (Aller *et al*, 2009; Singh & Settleman, 2010; Saxena *et al*, 2011). SLUG and SNAIL contribute to evading therapy‐induced apoptosis by interfering with p53 function, repression of the tumor‐suppressor *PTEN*, or upregulation of the pro‐survival protein BCL‐XL (Vega *et al*, 2004; Escriva *et al*, 2008; Kurrey *et al*, 2009; Wu *et al*, 2015; Cao *et al*, 2016).

Experimentally transformed HMLER breast cancer cells with increased EMT features display about 10‐fold increase in IC50 doses of many chemotherapeutics (Gupta *et al*, 2009; Singh *et al*, 2009; Singh & Settleman, 2010; Tulchinsky *et al*, 2019). In lineage tracing experiments, GFP‐labeled mesenchymal tumor cells in PyMT tumors display high resistance to cyclophosphamide treatment (Fischer *et al*, 2015). In non‐small‐cell lung cancer (NSCLC), activation of the AXL receptor tyrosine kinase in the course of EMT provides resistance to EGFR and PI3K inhibition, dependent on sustained KRAS activity (Singh *et al*, 2009; Sequist *et al*, 2011; Zhang *et al*, 2012; Byers *et al*, 2013; Tulchinsky *et al*, 2019). Furthermore, ZEB1 downregulation through HDAC class I inhibition or DNA demethylation resensitizes pancreatic cancer and osteosarcoma cells to chemotherapy (Meidhof *et al*, 2015; Ruh *et al*, 2021). Docetaxel‐resistance of lung adenocarcinoma cells and paclitaxel‐resistance of ovarian cancer cells can be reverted by blocking *ZEB1* expression, providing increased survival of lung metastasis‐bearing mice upon treatment (Ren *et al*, 2013; Sakata *et al*, 2017). In BRAF‐mutant melanoma, ZEB1 is sufficient and only partly compensated by TWIST to promote an undifferentiated p75^high^ neural crest stem cell‐like state that is characterized by resistance to MAPK and BRAF inhibitors (Richard *et al*, 2016; Shaffer *et al*, 2017; Rambow *et al*, 2018). In contrast, TWIST and SNAIL dampen the response to gemcitabine in an autochthonous mouse model of pancreatic cancer as *Snai1* and *Twist1* knockout results in prolonged survival in gemcitabine‐treated tumor mice (Zheng *et al*, 2015). Similarly, in NSCLC TWIST inhibition leads to sensitization to tyrosine kinase inhibitors of mutant EGFR (Yochum *et al*, 2019). In glioblastoma, the EMT‐regulated shift toward a more stem‐cell‐like phenotype correlates with increased radioresistance. This is mediated by elevated STAT3 which is directly binding to the *SNAI2* promoter for transcriptional activation (Lin *et al*, 2018). Collectively, EMT induction, increase in cancer stem cell features, and acquisition of therapy resistance are strongly connected and EMT‐TFs are central players in orchestrating these processes.

### EMT induced by therapy

As outlined before, one feature of EMT is to provide resistance to several treatments, including chemo‐, radio‐, and immunotherapy. Of note, also the therapy itself can induce an EMT phenotype with more aggressive features that involves activation of EMT promoting pathways via TGFβ, NF‐κB, WNT, FGF, and EGF/HER2 (see below) (Terry *et al*, 2017; Redfern *et al*, 2018). Therapy‐induced selection of cells with EMT characteristics, eventually displaying gain of *ZEB1* and *VIM*, but repressed *CDH1*, *MIR200C* and *MIR205* expression, was observed *in vitro* after long‐term exposures to gemcitabine in BxPC3 pancreatic cancer cells and to Docetaxel in PC3 and DU‐145 prostate cancer cells (Wellner *et al*, 2009; Puhr *et al*, 2012). Similarly, withdrawal of BRAF and MEK inhibition results in an enhanced motility and invasion through reactivation of ERK1/2 and AKT/mTOR *in vitro* (Norz *et al*, 2015). Treatment of mice suffering from CRC liver metastasis, with a vascular disruptive agent leads to a transient accumulation of nuclear β‐catenin and upregulation of *ZEB1*, which reverts after treatment. This indicates that EMT induction might display adoption to cope with therapy‐induced cellular stress (Fifis *et al*, 2013; Terry *et al*, 2017; Redfern *et al*, 2018). Supporting this hypothesis, inhibition of factors involved in therapy‐induced DNA damage response (DDR), like CHK1 activation by ATR inhibitors, results in EMT and *ZEB1* upregulation in CRC cells, reduced phosphorylation of CHK1 and decreased sensitivity to ATR inhibition (Song *et al*, 2018; Zhang *et al*, 2018). Observations in patients support a clinical relevance of EMT‐associated pro‐survival stress adaptation, exemplified by analysis of post‐therapy specimens, e.g., in esophageal and breast cancer after chemotherapy and in CRC after radiotherapy, showing decreased E‐cadherin and elevated SNAIL and Vimentin levels (Creighton *et al*, 2009; Kawamoto *et al*, 2012; Hara *et al*, 2014).

### Metabolic reprogramming

Metabolic rewiring is one important feature of cancer cells to cope with the excessive needs for energy and nutrients, enabling unlimited proliferation in conditions of rather restricted oxygen supply. In support of the metastasis cascade, reprogramming of glucose, lipid, and amino acid metabolism is tethered to activation of the EMT program and orchestrated by core EMT‐TFs (Kang *et al*, 2019; Georgakopoulos‐Soares *et al*, 2020; Sun & Yang, 2020; Bergers & Fendt, 2021). Cancer cell‐mediated changes in metabolism are well described in glycolysis and triglyceride pathways, favoring the “Warburg effect”, i.e., energy‐inefficient glycolysis even under normoxic conditions (Sun & Yang, 2020). This is another example how cancer cells adopt a developmental program of enhanced aerobic glycolysis that favors EMT and migration in the embryo (Youssef & Nieto, 2020). Upregulation of glucose transporters GLUT1 and GLUT3 is further promoting glycolysis. It was demonstrated in NSCLC that the increase in GLUT3 correlates with poor prognosis and EMT activation, mediated by activation of ZEB1 (Masin *et al*, 2014). On the contrary, metabolic abnormalities, e.g., in the tricarboxylic acid (TCA) cycle, can facilitate EMT. For example, loss of fumarate hydratase (FH) leads to aberrant methylation of a miR‐200 family promoter region, resulting in enhanced expression of ZEB factors and EMT induction. Fumarate accumulation by repression of FH by the chromatin modifier LSH subsequently increases α‐ketoglutarate (α‐KG) levels. This affects IKKα‐dependent EMT by promoting IKKα binding to and activation of EMT‐associated gene promoters (He *et al*, 2016; Sciacovelli *et al*, 2016; Sciacovelli & Frezza, 2017; Georgakopoulos‐Soares *et al*, 2020). Interestingly, ZEB1 is essential to allow metabolic plasticity. ZEB1 deficient pancreatic cancer cell lines are incapable of compensatory increasing glycolysis when oxidative phosphorylation is blocked, highlighting poor glycolytic reserve (Krebs *et al*, 2017). A systematic approach to categorize metabolic rewiring during EMT focused on gene expression alterations in high grade malignancies and identified a mesenchymal metabolic signature of 44 genes. Upregulation of this group of genes is correlated with a mesenchymal phenotype and is controlled by TWIST1 in mammary epithelial cells (Shaul *et al*, 2014).

In addition to carbohydrate pathways, EMT‐associated changes in fatty acid, lipid and glycosphingolipid metabolisms are detected (Kang *et al*, 2019; Georgakopoulos‐Soares *et al*, 2020; Hua *et al*, 2020; Bergers & Fendt, 2021). The morphological changes of cancer cells during EMT are accompanied by alterations in membrane fluidity and lipid composition. This requires the elevation of available fatty acids and is achieved for example by increased expression of the fatty acid translocase CD36. The presence of CD36 promotes EMT in HCC and is crucial for metastatic colonization of oral cancer (Nath *et al*, 2015; Pascual *et al*, 2017; Bergers & Fendt, 2021). Elevated free fatty acids (FFAs) lead to increased SRC and MMP9 activity that accelerates metastasis (Gubelmann *et al*, 2014; Jiang *et al*, 2020). In addition, the synthesis, storage and use of long‐chain polyunsaturated fatty acids (PUFA) is elevated in the therapy‐resistant EMT cell state. Consequently, the cells become dependent on elimination of resultant reactive lipid peroxides by Glutathione peroxidase 4 (GPX4) to evade the iron‐dependent cell death pathway ferroptosis (Viswanathan *et al*, 2017). In summary, these analyses are a few examples demonstrating the reciprocal connection of EMT, cell plasticity, and metabolic reprogramming and how they synergize toward malignant progression (Fig 3).

### EMT and immune evasion

During tumor progression, cancer cells develop a variety of strategies to withstand immunosurveillance and adaptive anti‐tumor immunity that is induced by tumor neo‐antigens. Most commonly pursued strategies include hiding the neo‐antigen spectrum and formatting of an immunosuppressive environment, e.g., by hijacking negative immunological feedbacks referred to as “immune checkpoints” (Terry *et al*, 2017; Kudo‐Saito *et al*, 2021). Interestingly, these unwanted processes often occur in strong correlation with EMT, and EMT‐TFs have repeatedly been shown to co‐regulate them directly or indirectly (Terry *et al*, 2017; Dongre & Weinberg, 2019; Jiang & Zhan, 2020; Kudo‐Saito *et al*, 2021). For instance, defects in the immunoproteasome, that generates peptides for antigen presentation, were associated with a more mesenchymal phenotype in NSCLC (Tripathi *et al*, 2016). A study using the PyMT mouse model of breast cancer demonstrated a remarkable anti‐correlation of mesenchymal expression profiles and MHC I expression (Dongre *et al*, 2017). Thus, human leukocyte antigen (HLA) class I complexes and major histocompatibility complex I (MHC I), required for antigen presentation, can apparently be deregulated by EMT‐related mechanisms. Furthermore, utilizing breast cancer models *in vitro* and *in vivo* demonstrated that more mesenchymal tumor cells promote the generation and recruitment of regulatory T cells (T_regs_) (Joffroy *et al*, 2010; Akalay *et al*, 2013; Dongre *et al*, 2017), which are an integral part of immune checkpoints able to inhibit cytotoxic T cells (CTL). In this regard, one of such intrinsic “brake” regulatory mechanisms of CTLs is activated by binding of their programmed cell death receptor (PD1) to its ligand PD‐L1 provided by T_regs_ and myeloid antigen presenting cells. Tumor cells can hijack these immune checkpoints mainly by upregulation of *CD274* (PD‐L1), which is strongly promoted by the EMT program (Zou *et al*, 2016; Terry *et al*, 2017; Jiang & Zhan, 2020; Kudo‐Saito *et al*, 2021). In NSCLC, PD‐L1 is activated by deregulation of the ZEB1/miR‐200 axis in more mesenchymal cells (Chen *et al*, 2014; Jung *et al*, 2020). This regulation is likely independent of a general EMT program, since ZEB1 loss cannot be compensated by SNAIL, SLUG, or TWIST (Noman *et al*, 2017). Kudo‐Saito showed that *SNAI1* expression in melanoma promotes induction of T_regs_ via Thrombospondin1 activation that results in resistance to immunotherapy (Kudo‐Saito *et al*, 2009). Based on these findings, assessing the EMT signatures of, e.g., bladder cancer and melanoma may help to select patients for immune checkpoint blockers (Hugo *et al*, 2016; Kardos *et al*, 2016). In summary, EMT cells evade T‐cell anti‐tumor immunity either via downregulation of antigen presentation or activation of immune checkpoints.

### EMT and DNA integrity

Genomic instability is a common feature in cancer with evidence of contribution of EMT (Nieto *et al*, 2016; Yang *et al*, 2020) and has frequently been observed in pre‐malignant mesenchymal cells *in vitro* as well as in tumors (Massague, 2012; Comaills *et al*, 2016; Caramel *et al*, 2018; Wang *et al*, 2019a). Although EMT stimuli are normally anti‐proliferative, some cancer cells maintain cell doubling, perhaps at the expense of genome stability (Omabe *et al*, 2021). The involved mechanisms overriding the anti‐proliferative signals and the generation of genomic aberrations are poorly understood. Usually, DNA repair, cell cycle checkpoints, senescence and cell death in response to endogenous (e.g., during DNA replication) and exogenous DNA damages (e.g., during chemotherapy) are orchestrated by the DDR, which is a conserved, multifactorial and context‐dependent signaling network (Halazonetis *et al*, 2008; Omabe *et al*, 2021). Recent evidence suggests that EMT‐TFs differentially affect the DDR. TWIST promotes chromosomal instability (CIN) by repressing mitotic checkpoint factors in colorectal cancer (CRC), whereas SLUG facilitates homologous recombination‐directed DNA repair (HDR) during replication stress in mammary epithelial cells (Gross *et al*, 2019; Khot *et al*, 2020). Hence, regulation of the DDR by EMT‐TFs may influence cancer treatment. In breast cancer cells, ZEB1 promotes radioprotection by driving the expression of the apical DDR kinase ATM via complex formation with p300 and PCAF at the *ATM* promoter as well as by stabilizing the mediator kinase CHK1 via sequestration of its deubiquitinase USP7 (Zhang *et al*, 2014; Zhang *et al*, 2018). In triple‐negative breast cancer, ZEB1 was shown to directly repress *POLQ* (Polθ) expression to prevent usage of the highly mutagenic alternative end joining (a‐EJ or microhomology‐mediated end joining) for the repair of endogenously occurring DSBs, thereby supporting the maintenance of genomic stability (Prodhomme *et al*, 2021). Intriguingly, during breast cancer development, the accumulation of CIN is apparently prevented, if oncogene activation had taken place in mammary stem cells, engaging a ZEB1‐driven preemptive program with upregulation of the anti‐oxidant MSRB3. This mitigates oncogene‐induced replication stress to overcome oncogene‐induced senescence (OIS) in *TP53* wild‐type cells (Halazonetis *et al*, 2008) (Morel *et al*, 2017). Along that line, SNAIL and TWIST regulate p53 and RB‐dependent pathways to modulate OIS (Ansieau *et al*, 2008; Puisieux *et al*, 2014). In addition, SNAIL and SLUG alter the downstream DDR‐driven cell death in MCF7 breast cancer cells by repressing pro‐apoptotic effectors (Kajita *et al*, 2004). In summary, tumor cells utilize specific DDR‐related functions of EMT‐TFs to evade oncosuppressive mechanisms.

**Box 1 Signaling pathways activating EMT in cancer**

**TGFβ**

**Figure 4:**
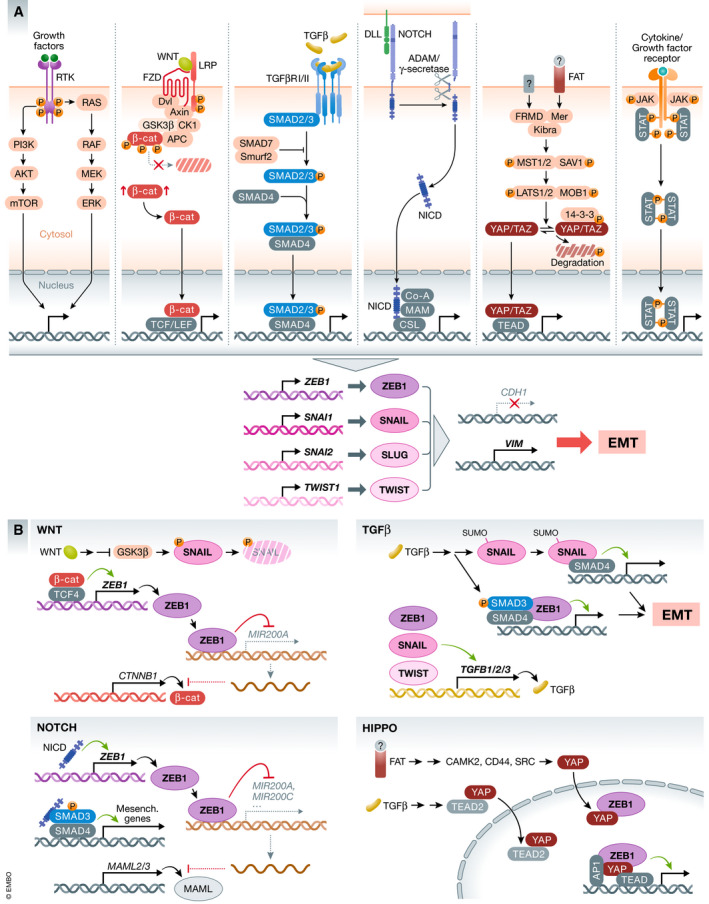
Signaling pathways and their crosstalk during EMT induction (A) Canonical receptor tyrosine kinase pathways activated by EGF/EGFR, PDGF/PDGFR, HGF/c‐Met and others, Wnt, TGFβ, Notch, Hippo, and Cytokine signaling (e.g., IL6, TNFα) and their nuclear effectors are shown, which all promote expression of core EMT‐TFs. Activation of EMT‐TFs results in execution of the EMT program, exemplified by regulated *CDH1* and *VIM* expression. EMT is promoted by crosstalk of signaling pathways on multiple levels already in the cytoplasm (not shown). (B) Examples of EMT gene regulation of individual pathways and input from other signal transduction pathways. Effects on direct gene activation and repression are shown by green arrows and red block connections, respectively. For details, see text.

TGFβ is the major extrinsic signal of EMT in tumors, secreted by many cell types in the TME, but the role of TGFβ signaling in tumor initiation and progression is very complex as it shows a biphasic function with opposing effects. During tumor initiation, TGFβ promotes differentiation, cell cycle arrest, senescence and apoptosis in pre‐malignant epithelial cells whereas during later stages TGFβ acts as inducer of EMT and promotes metastasis (Massague, 2012; Seoane & Gomis, 2017; Ramachandran *et al*, 2018; Derynck *et al*, 2021; Liu *et al*, 2021) (Fig 4A). Moreover, TGFβ acts in a very context‐dependent manner in crosstalk with other pathways, including Hippo, WNT, Notch, Hedgehog, ERK, p38 MAPK, PI3K/AKT and RHO‐like GTPases to enhance their function (Bakin *et al*, 2000; Zavadil & Bottinger, 2005; Hao *et al*, 2019; Derynck *et al*, 2021; Liu *et al*, 2021) (Fig 4A). In transformed mammary epithelial cells, execution of TGFβ‐mediated EMT depends on autocrine WNT activity and inhibition of these autocrine cues reduces tumorigenicity and metastasis *in vivo* (Scheel *et al*, 2011). TGFβ induces mesenchymal genes like *VIM* and *FN1* as well as EMT‐TFs *SNAI1*, *SNAI2*, *TWIST1* and *ZEB1* which form a feedback loop by activating TGFβ ligand expression to self‐enforce and maintain TGFβ signaling (Dongre & Weinberg, 2019). Initial TGFβ receptor activation in breast or skin cancer results in transcriptional activation of *SNAI1*, combined with sumoylation of SNAIL at K234 fostering SMAD/SNAIL complex formation that promotes EMT (Hoot *et al*, 2008; Vincent *et al*, 2009; Gudey *et al*, 2017) (Fig 4B). Similarly, ZEB proteins have SMAD‐interacting domains and TGFβ, in crosstalk with MAPK signaling, utilizes SMAD3/SMAD4/ZEB1/2 complexes to regulate EMT‐related gene expression (Postigo, 2003; Shirakihara *et al*, 2007). Very strikingly, in pancreatic cancer, TGFβ‐mediated EMT depends almost exclusively on ZEB1, since *Zeb1* knockout in KPC cells blocks more than 90% of TGFβ‐induced alterations in gene expression, despite of *Snai1* upregulation during TGFβ treatment (Krebs *et al*, 2017).

**Notch**

Like TGFβ, the Notch pathway is important for differentiation processes during development that in part involve EMT by *SNAI1* and *SNAI2* (Timmerman *et al*, 2004; Niessen *et al*, 2008). In cancer, a Notch‐dependent EMT program is in part executed by cooperating with TGFβ signaling (Yuan *et al*, 2014; Bray, 2016). Activation of the Notch pathway by Delta/Jagged ligands results in processing and release of the Notch ICD transducer that in turn interacts with SMADs, in addition to canonical RBP/J cofactor binding (Fig 4A and B). This crosstalk is essential for mesenchymal gene activation and regulates bone metastasis in NSCLC (Blokzijl *et al*, 2003; Xie *et al*, 2012; Liu *et al*, 2014a; Yuan *et al*, 2014). While in NSCLC Notch3 is crucial for invasion and EMT by directly regulating *ZEB1* transcription, in stratified squamous epithelia Notch3 promotes terminal differentiation. Oncogene‐induced *NOTCH1* in SCC is activating *ZEB1* expression, which in turn downregulates *NOTCH3* to promote stem cell features and block differentiation (Liu *et al*, 2014a; Natsuizaka *et al*, 2017). Moreover, the ZEB1/miR‐200 feedback loop further modulates Notch signaling activity as downstream Notch components including MAML2/3 are miR‐200 targets, resulting in enhanced Notch activation upon *ZEB1* induction (Brabletz *et al*, 2011) (Fig 4B). Pancreatic cancer metastasis is promoted by a Notch‐dependent inflammatory feedback loop that involves recruitment and activation of M2‐like tumor‐promoting macrophages and EMT via IL6/STAT3 signaling (Geng *et al*, 2021).

**Hippo/YAP/TAZ**

Deregulation of the Hippo pathway was shown to promote tumorigenesis as well (Moroishi *et al*, 2015; Ansari *et al*, 2019) (Fig 4A). YAP/TAZ overexpression and activation is crucial to mediate EMT and cell plasticity in skin cancer and is sufficient to induce EMT in MCF10A non‐cancerous mammary epithelial cells. YAP was shown to cooperate with KRAS for progression of pancreatic cancer and TAZ is a promoter of proliferation, EMT and stemness in breast and oral cancer (Zhang *et al*, 2009; Shao *et al*, 2014; Li *et al*, 2015; Debaugnies *et al*, 2018). The function of YAP/TAZ is dependent on the TEAD‐binding domain and in breast cancer, TEAD2 is key to orchestrate EMT by regulating nuclear localization of YAP and TAZ (Lamar *et al*, 2012; Diepenbruck *et al*, 2014). In the very aggressive subgroup of Claudin‐low breast cancer, YAP and ZEB1 interact to drive expression of a common target gene subset to promote EMT and metastasis (Lehmann *et al*, 2016). Moreover, YAP forms a TEAD/YAP/AP1 ternary complex to regulate activity of distal enhancers, whereas ZEB1 and YAP are part of a multimeric complex with AP1 factors independent of direct ZEB1 DNA binding to drive gene expression on a genome‐wide level (Zanconato *et al*, 2015; Feldker *et al*, 2020) (Fig 4B). Wang and colleagues demonstrated that in breast cancer, also TWIST is involved in YAP/TAZ‐mediated EMT orchestrated by TWIST‐dependent activation of the GPCR member *PAR1* (Wang *et al*, 2020).

**WNT**

The role of WNT/β‐catenin signaling in tumorigenesis and metastasis is highlighted by mutations of APC that preclude the control of the signaling competent cytoplasmic pool of β‐catenin (Fig 4A). Moreover, the WNT target G‐coupled receptor LGR5 labels stem cells in several organs as well as cancer stem cells in tumors (Clevers, 2006). Interestingly, LGR5 expression and WNT activity are crucial for disease progression, induction of EMT, stemness and metastasis, which is not provided by LGR5‐negative tumor cells and depletion of Lgr5^+^ cells in mice blocks metastasis (de Sousa e Melo *et al*, 2017), though a Lgr5^‐^ state is detected in circulating CSCs (Fumagalli *et al*, 2020). In breast cancer, reduction in WNT signaling by addition of secreted negative regulators of the pathways, like DKK1 or SFRP1 can promote reduction in migration and invasion, whereas inhibition of SFRP1, and thus elevated WNT signaling, can support an EMT phenotype and sensitize to TGFβ‐induced EMT (Gauger *et al*, 2011; Scheel *et al*, 2011). Intracellularly, the WNT‐induced block of GSK3β is not only promoting β‐catenin cytoplasmic accumulation, but also stabilizes SNAIL (Zhou *et al*, 2004). *ZEB1* is transcriptionally activated via β‐catenin/TCF4 binding to the *ZEB1* promoter (Sanchez‐Tillo *et al*, 2011). Furthermore, increased ZEB1 in turn represses *MIR200A* expression which leads to de‐repression of *CTNNB1* (β‐catenin) thereby fueling this feedback loop (Su *et al*, 2012; Liu *et al*, 2013) (Fig 4B).

**RTK signaling (induced by, e.g., EGF, FGF, IGF, HGF and PDGF)**

Receptor tyrosine signaling has pleiotropic functions in proliferation and differentiation during development and homeostasis as well as in cancer initiation and progression. Growth factors like EGF, FGF, HGF and PDGF act via their cognate receptors and promote EMT either in support of TGFβ or alone via MAPK/ERK, PI3K/AKT and mTORC signaling (Lamouille & Derynck, 2007; Lamouille *et al*, 2012; Dongre & Weinberg, 2019) (Fig 4A). The importance of RTK signaling is best described by the key function of RAS (*KRAS*, *HRAS*, *NRAS*) and *BRAF* oncogenes. One of them is mutated in most cancer types, leading to continuous pathway activation. *SNAI1* and *SNAI2* are direct targets of mutant KRAS and BRAF activities and *ZEB1*/*2* is regulated downstream by ERK2, but not ERK1 (Shin *et al*, 2010; Makrodouli *et al*, 2011). EGF induces EMT by upregulation of *SNAI1*, *TWIST1* and *ZEB1* in different cell lines through MAPK/ERK as well as JNK/STAT3 activation (Lu *et al*, 2003). In particular, EGF is cooperating with other signaling pathways, e.g., IL6R/STAT3 and TGFβ, resulting in *CDH1* downregulation and enhanced cell motility and invasion (Lo *et al*, 2007; Tian *et al*, 2007; Uttamsingh *et al*, 2008; Colomiere *et al*, 2009). Phosphorylation of SMAD2/3 and nuclear co‐localization with SNAIL was induced by activation of EGFR in MCF7 and MDA‐MB‐231 breast cancer cells (Kim *et al*, 2016). EGF‐induced EMT seems to require the function of the RNA‐binding protein Musashi2 (MSI2) by interaction with ZEB1 to facilitate ZEB1‐ERK/MAPK signaling in pancreatic cancer (Sheng *et al*, 2020). Similar examples exist for activation of FGFR, IGFR, PDGFR and c‐Met (HGF receptor) (Grotegut *et al*, 2006; Yang *et al*, 2006; Graham *et al*, 2008; Devarajan *et al*, 2012; Zhu *et al*, 2016; Maehara *et al*, 2017; Zheng *et al*, 2019).

**Cytokines and hypoxia**

Several inflammatory cytokines, produced by cells in the TME, are contributing to cellular plasticity and an invasive phenotype (Fig 4A). Most prominently TNFα and IL6, but also IL1β have critical functions and promote stemness, invasion and regulation of genes involved in EMT mediated by SNAIL, TWIST and ZEB1 as demonstrated in colon, breast and renal cell carcinoma (Bates & Mercurio, 2003; Sullivan *et al*, 2009; Ho *et al*, 2012; Li *et al*, 2012; Miao *et al*, 2014; Ieda *et al*, 2019). IL6 signals through STAT3 that directly activates *SNAI1* and *ZEB1* expression, whereas TNFα facilitates TGFβ and PI3K/AKT signaling that inhibits GSK3β (Bates & Mercurio, 2003; Sullivan *et al*, 2009; Ho *et al*, 2012; Miao *et al*, 2014) (Fig 4A). Interestingly, an autocrine feedback loop is generated in tumor cells to enhance an inflammatory phenotype, since SNAIL and ZEB1/2 are able to activate *IL6* expression in head and neck squamous carcinoma and breast cancer (Lyons *et al*, 2008; Katsura *et al*, 2017).

In addition to growth factors, chemokines and cytokines, hypoxia and HIF1α are well known to induce EMT as well (Hapke & Haake, 2020). In this scenario, EMT is induced by inefficient neo‐angiogenesis and deprivation of pericytes in the tumor (Hapke & Haake, 2020). Activated HIF1α or HIF2α directly or indirectly regulate *SNAI1*, *TWIST1* and *ZEB1* expression which fosters stemness and metastasis formation in bladder, ovarian, gastric and breast cancer (Yang *et al*, 2008; Cooke *et al*, 2012; Liu *et al*, 2017; Hapke & Haake, 2020; Zhang *et al*, 2020).

 

In summary, data that have been collected over decades provide a comprehensive picture in which EMT in tumorigenesis and metastasis is induced by integration of many pathways and growth factors. Moreover, synergism and the crosstalk of several signaling networks highlight that cellular plasticity in epithelial tumor cells renders the promotion of a cancer stem cell‐like, immune evading, multi‐resistant, invasive phenotype, able to adapt to changing conditions in the tumor and the metastatic niches. However, detailed mechanistic insight is still lacking to understand the complex crosstalks of signaling pathways that drive EMT and metastasis.

## The tumor microenvironment: Stroma‐tumor crosstalk to foster EMT

**Figure 5:**
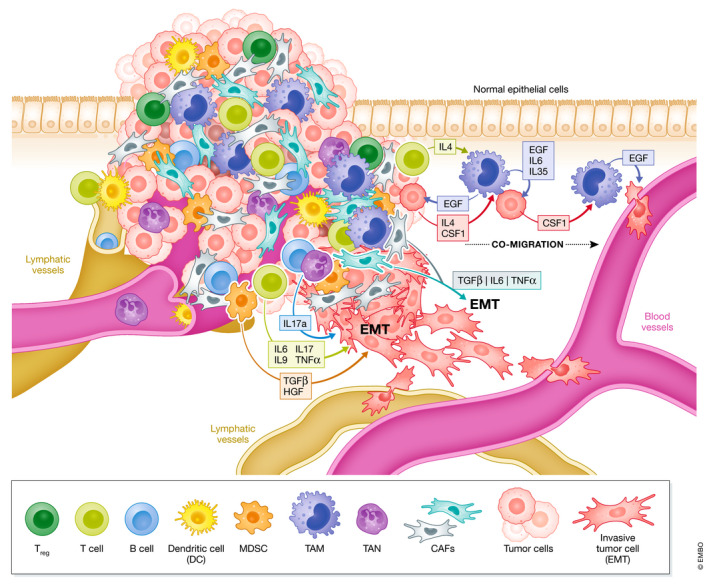
Cell types in the tumor microenvironment and contribution to EMT Cells of the hematopoietic lineage, fibroblasts and others contribute to the TME. Exemplified modes of action and cytokines secreted from individual cell types are depicted which promote EMT, migration, invasion, and intravasation of tumor cells into blood vessels. EMT induction and co‐migration can be induced by TAMs and specific lymphocyte/tumor cell crosstalk.

The TME is the source of many different signaling cues that have been implicated in EMT induction, including TGFβ, WNT, RTK, Hippo, Notch and cytokine signaling (Box 1, Fig 4). The TME consists of extracellular matrix and non‐transformed cells that are recruited to the tumor and adopt a variety of abnormal phenotypes instructed by tumor/stroma crosstalk. Cell types include fibroblasts, cells of the immune system, endothelial cells, adipocytes and neuronal cells (Fig 5). The complex crosstalk of the entity of these cell types and the ECM is providing pro‐ and anti‐tumorigenic functions. Stroma composition has a strong impact on tumor aggressiveness and carries prognostic value (Hanahan & Weinberg, 2011; Jin & Jin, 2020). Moreover, individual stromal cell types are contributing to EMT and metastasis.

### CAFs

Cancer‐associated fibroblasts (CAFs) display a very heterogenous population generated by reprogramming and expansion of either local tissue‐resident or recruited fibroblasts from the periphery, including transdifferentiation of adipocytes, pericytes, monocytes, and mesenchymal stem cells (LeBleu & Kalluri, 2018; Sahai *et al*, 2020). CAFs can be categorized into different subpopulations with specific functions and spatial organization (Lambrechts *et al*, 2018; Elyada *et al*, 2019; Sahai *et al*, 2020). They serve as a primary source of EMT‐inducing signaling molecules, like IL6, TNFα and TGFβ, which promote a mesenchymal phenotype of tumor cells (Fig 5). Consequently, as an example they increase chemoresistance in NSCLC, regulate metastasis in ovarian cancer and promote oncogenic potential of adjacent epithelia in gastric squamous cell carcinoma (Bhowmick *et al*, 2004; Yu *et al*, 2014; Calon *et al*, 2015; Shintani *et al*, 2016; Zhao *et al*, 2017; Goulet *et al*, 2019). As demonstrated for prostate cancer, the entry into TGFβ‐mediated EMT initiated by CAFs is dependent on changes in DNA methylation by DNMTA3A, silencing epithelial genes like *CDH1* and *GRHL2* (Pistore *et al*, 2017). TGFβ secretion by CAFs and EMT signatures in CRC are correlated with poor prognosis and promote metastasis (Calon *et al*, 2015; Tauriello *et al*, 2018). Interestingly, WNT signaling is involved in orchestrating CAF diversity and crosstalk to CRC tumor cells. In a subcutaneous organoid model, SFRP1 secretion induces a switch from contractile myCAFs to inflammatory iCAFs, accompanied by increasing EMT signatures in tumor cells (Mosa *et al*, 2020). Moreover, CAFs can impinge on anti‐tumor immunity and on success of anti‐CSFR1 immunotherapy (Kumar *et al*, 2017; Tauriello *et al*, 2018), supported by the finding that collagen 1 (COL1) deposition by myCAFs is crucial to augment an immunosuppressive TME (Chen *et al*, 2021).

### TAMs

Macrophages in the TME are contributing to EMT on multiple levels. In a simplified model, macrophage‐tumor crosstalk generates a variety of tumor‐associated macrophage (TAM) phenotypes, with an M1‐like state promoting elimination of immunogenic tumor cells and a contrasting pro‐tumorigenic M2‐like state (Gonzalez *et al*, 2018). In crosstalk with other cell types and in the presence of TGFβ, metastasis is induced by CD4^+^ T‐cell derived IL4 and macrophage‐derived WNT7B/EGF as well as CSF1 from tumor cells. These ligands induce co‐migration of tumor cells and macrophages toward blood vessels. TAMs near blood vessels create “the tumor microenvironment of metastasis” enabling tumor cell intravasation (Joyce & Pollard, 2009; Noy & Pollard, 2014; Gonzalez *et al*, 2018) (Fig 5). TAMs can also directly express and secrete TGFβ and cytokines to induce EMT. In F9 teratocarcinoma, NMuMG cells and hepatocellular carcinoma (HCC), intratumoral or cocultured macrophages are secreting TGFβ to promote EMT (Bonde *et al*, 2012; Fan *et al*, 2014). In CRC, EMT is further accelerated by TNFα‐secreting macrophages, inducing p38 MAPK signaling in tumor organoids (Bates & Mercurio, 2003). Moreover, in renal clear cell carcinoma, NSCLC, colon and breast cancer IL6 and IL35 are produced by macrophages and promote EMT by prostaglandin/β‐catenin and JAK2‐STAT6‐GATA3 signaling, respectively (Zhang *et al*, 2019b; Sun *et al*, 2020) (Che *et al*, 2017; Lee *et al*, 2018).

### MDSCs

Myeloid‐derived suppressor cells (MDSCs) are a heterogenous cell population originating from neutrophils and monocytes. They are expanding in cancer patients and are involved in immune cell suppression, specifically of T cells. Accordingly, they promote tumor cell immune escape and block cytotoxic T‐cell activity (Gabrilovich & Nagaraj, 2009; Gabrilovich, 2017). Moreover, they directly induce EMT via release of different growth factors and cytokines, including TGFβ and HGF, as well as the high mobility group protein B1 (HMGB1) which acts through toll‐like receptors (TLRs) and leads to induction of *SNAI1*, *MMP7* and *NFKB1* (NF‐κB) (Trovato *et al*, 2020) (Fig 5). At the primary tumor, specific monocytic *CXCR2*‐expressing MDSCs (mMDSCs) promote EMT and they are recruited by breast cancer cells via secretion of CXCL1/2, whereas MET at pulmonary metastatic sites is induced by granulocytic MDSCs (gMDSCs) (Ouzounova *et al*, 2017; Zhu *et al*, 2017; Taki *et al*, 2018; Trovato *et al*, 2020). Of note, MDSCs become activated and expand upon operational stress during primary breast cancer resection and start to secrete TGFβ, VEGF and IL10 to promote lung metastasis in a 4T1 orthotopic breast cancer model (Ma *et al*, 2019).

### TANs

Neutrophils are promoting metastasis at the pre‐metastatic niche in lungs and liver by suppressing T cells (Gonzalez *et al*, 2018). In addition, they are involved in promoting EMT at the primary site. Akin to TAMs, tumor‐associated neutrophils (TANs) can polarize between extreme states “N1” TANs with anti‐tumor and pro‐immune functions, induced by IFN‐β, and “N2” TANs with pro‐tumorigenic and pro‐metastatic functions. “N2” TANs are induced by TGFβ (Gonzalez *et al*, 2018; Masucci *et al*, 2019; Wu & Zhang, 2020). Neutrophils in the stroma of gastric cancer (GC) produce IL17a and CXCL5 both promoting EMT, indicated by upregulation of *VIM* and *ZEB1*, whereas *CDH1* is repressed (Fig 5). Anti‐IL17a treatment *in vitro* blocks EMT in neutrophil‐GC co‐cultures (Li *et al*, 2019; Mao *et al*, 2020; Wu & Zhang, 2020). In MCF7 breast cancer cells, EMT is induced by TIMP‐1 expression secreted by neutrophils. TAN‐MCF7 interaction creates an enforcing feedback‐loop as MCF7 cells with a more mesenchymal phenotype activate *TIMP‐1* expression in neutrophils by CD90 during direct cell contact. Blocking CD90 reduces the number of metastasis in a 4T1 orthotopic injection model in mice (Wang *et al*, 2019b; Wu & Zhang, 2020).

### T cells

CD4^+^ and CD8^+^ T cells are the most prominent effectors of anti‐tumor immune response. High levels of T‐cell infiltration usually correlate with better prognosis; however, their activity is dependent on immune regulatory and suppressive mechanisms including T_reg_ activities (Joyce & Pollard, 2009; Gonzalez *et al*, 2018). Moreover, some examples demonstrate direct contribution of T cells to EMT. In the presence of isolated effector CD4^+^ T cells, pancreatic cancer cells show upregulation of *VIM*, *L1CAM* and *ZEB1*, combined with a more migratory phenotype, which was reverted by blocking IL6 and TNFα (Goebel *et al*, 2015). In breast cancer, also CD8^+^ cytotoxic T cells induce EMT, specifically during relapse and loss of a HER2/neu antigen‐mediated response, resulting in a more stem cell‐like phenotype with increased therapy resistance (Kmieciak *et al*, 2007; Santisteban *et al*, 2009). In lung cancer, specific T‐lymphocyte subsets induce EMT in co‐culture experiments via IL9 and IL17 secretion and block of IL9/IL17 reduced EMT and metastasis (Salazar *et al*, 2020) (Fig 5).

## Therapeutic options to target EMT

As outlined in the previous chapters, various aspects of EMT are contributing to poor outcome, including resistance to treatment. Since therapeutic intervention with standard care, targeted therapy, irradiation, and surgery is often promoting EMT, major benefit is expected from therapeutic approaches that prevent or reverse the fatal effects of EMT. Standard treatment for targeting differentiated highly proliferative cancer cells combined with specific therapy to either block/revert an EMT/stemness/therapy‐resistant state or to target specific, yet unidentified, vulnerabilities of such exquisitely plastic cells seems advantageous. Several molecules to target EMT have been or are currently in clinical trials whereas others are already in use. These include blocking of upstream pathways which promote tumorigenesis also beyond EMT by ligand‐neutralizing antibodies, decoy receptors or inhibitors to block TGFβ, NF‐κB, EGFR, c‐MET, WNT and Notch signaling (comprehensively reviewed in (Redfern *et al*, 2018; Dudas *et al*, 2020; Jonckheere *et al*, 2021).

Due to difficulties in targeting transcription factors, i.e., EMT‐TFs, directly, another promising approach is to apply modified synthetic miRNAs that interfere with EMT‐TFs on a post‐transcriptional level. Preclinical data demonstrate that miR‐34 and miR‐200s administration will attenuate SNAIL and ZEB1 protein levels, respectively, and inhibit stemness and metastasis (Pecot *et al*, 2013; Cortez *et al*, 2014). Liposomal miR‐34 (MRX34) is already used in clinical trials with effects on tumor growth and metastasis (Zhang *et al*, 2019a).

Another interesting approach to block cell plasticity and to resensitize tumor cells to standard therapy is to induce re‐ or transdifferentiation. Interfering with chromatin remodeling by using HDAC inhibition or DNA‐demethylating agents has been proven valid methods to induce differentiation. In pancreatic cancer, the application of the HDAC class I inhibitor mocetinostat results in de‐repression of ZEB1‐inhibiting miRNAs, ZEB1 downmodulation, MET, and resensitization to gemcitabine in mice (Meidhof *et al*, 2015). Reactivating expression of silenced genes has a similar effect in osteosarcoma. Demethylation of the imprinted *DLK‐DIO3* locus by 5‐Azacitidine reactivates expression of ZEB1‐silencing miRNAs, induces osteogenic and adipogenic differentiation, and sensitizes to doxorubicin treatment (Ruh *et al*, 2021). The idea of transdifferentiation was nicely demonstrated by Ishay‐Ronen *et al*, showing that EMT‐derived breast cancer cells can be differentiated into post‐mitotic functional adipocytes by rosiglitazone *in vivo*, resulting in reduced metastasis (Ishay‐Ronen *et al*, 2019). Another group of molecules that induce re‐differentiation and drug sensitivity includes compounds generated for different purposes, like metformin, used for type2 diabetes, or herbal components, like curcumin (Kothari *et al*, 2014; Dudas *et al*, 2020; Jonckheere *et al*, 2021). Metformin leads to reduction in SNAIL, TWIST, and ZEB1 levels, and curcumin inhibits EMT by downregulation of NF‐κB/SNAIL in breast cancer and reactivation of epithelial miRNAs (Kothari *et al*, 2014; Dudas *et al*, 2020).

With all these promising approaches to block and reverse EMT, it is important to consider potential adverse effects. Although EMT is harmful during tumorigenesis and EMT‐TFs contribute to poor prognosis, they play very important physiological roles. EMT is involved in wound closure and repair after injury and EMT‐TFs have important functions in stem cell homeostasis and differentiation, e.g., in melanocytes, muscle, bone and the nervous system, as well as in innate and adaptive immune responses. Accordingly, global interference with the context‐dependent favorable actions of EMT‐TF by special therapies may well affect normal tissue homeostasis. Likewise, miRNA‐based applications will simultaneously regulate other targets with unpredicted outcome. On the contrary, therapeutic intervention that favors MET, might promote colonization and metastasis of circulating tumor cells, leading to opposing effects. Hence, a very careful evaluation and examination of adverse effects is needed to promote such approaches in the clinic. Moreover, we are only beginning to understand the complex nature of the multi‐tool EMT in cancer and integration of aspects of multiple pathways and of TME diversity are the main challenges toward a more comprehensive understanding of metastasis for identifying novel treatment approaches.

## Conflict of interest

The authors declare that they have no conflict of interest.

## References

[embj2021108647-bib-0001] Aceto N, Bardia A, Miyamoto D, Donaldson M, Wittner B, Spencer J, Yu M, Pely A, Engstrom A, Zhu H*et al* (2014) Circulating tumor cell clusters are oligoclonal precursors of breast cancer metastasis. Cell 158: 1110–1122 25171411 10.1016/j.cell.2014.07.013 PMC4149753

[embj2021108647-bib-0002] Ahmed N, Maines‐Bandiera S, Quinn MA, Unger WG, Dedhar S, Auersperg N (2006) Molecular pathways regulating EGF‐induced epithelio‐mesenchymal transition in human ovarian surface epithelium. Am J Physiol Cell Physiol 290: C1532–1542 16394028 10.1152/ajpcell.00478.2005

[embj2021108647-bib-0003] Aiello NM, Maddipati R, Norgard RJ, Balli D, Li J, Yuan S, Yamazoe T, Black T, Sahmoud A, Furth EE*et al* (2018) EMT subtype influences epithelial plasticity and mode of cell migration. Dev Cell 45: 681–695.e4 29920274 10.1016/j.devcel.2018.05.027 PMC6014628

[embj2021108647-bib-0004] Aigner K, Dampier B, Descovich L, Mikula M, Sultan A, Schreiber M, Mikulits W, Brabletz T, Strand D, Obrist P*et al* (2007) The transcription factor ZEB1 (deltaEF1) promotes tumour cell dedifferentiation by repressing master regulators of epithelial polarity. Oncogene 26: 6979–6988 17486063 10.1038/sj.onc.1210508 PMC2899859

[embj2021108647-bib-0005] Akalay I, Janji B, Hasmim M, Noman MZ, André F, De Cremoux P, Bertheau P, Badoual C, Vielh P, Larsen AK*et al* (2013) Epithelial‐to‐mesenchymal transition and autophagy induction in breast carcinoma promote escape from T‐cell‐mediated lysis. Cancer Res 73: 2418–2427 23436798 10.1158/0008-5472.CAN-12-2432

[embj2021108647-bib-0006] Aller SG, Yu J, Ward A, Weng Y, Chittaboina S, Zhuo R, Harrell PM, Trinh YT, Zhang Q, Urbatsch IL*et al* (2009) Structure of P‐glycoprotein reveals a molecular basis for poly‐specific drug binding. Science 323: 1718–1722 19325113 10.1126/science.1168750 PMC2720052

[embj2021108647-bib-0007] Ansari D, Ohlsson H, Althini C, Bauden M, Zhou Q, Hu D, Andersson R (2019) The hippo signaling pathway in pancreatic cancer. Anticancer Res 39: 3317–3321 31262852 10.21873/anticanres.13474

[embj2021108647-bib-0008] Ansieau S, Bastid J, Doreau A, Morel A‐P, Bouchet BP, Thomas C, Fauvet F, Puisieux I, Doglioni C, Piccinin S*et al* (2008) Induction of EMT by twist proteins as a collateral effect of tumor‐promoting inactivation of premature senescence. Cancer Cell 14: 79–89 18598946 10.1016/j.ccr.2008.06.005

[embj2021108647-bib-0009] Arnoux V, Nassour M, L'Helgoualc'h A, Hipskind RA, Savagner P (2008) Erk5 controls Slug expression and keratinocyte activation during wound healing. Mol Biol Cell 19: 4738–4749 18716062 10.1091/mbc.E07-10-1078 PMC2575153

[embj2021108647-bib-0010] Bakin AV, Tomlinson AK, Bhowmick NA, Moses HL, Arteaga CL (2000) Phosphatidylinositol 3‐kinase function is required for transforming growth factor beta‐mediated epithelial to mesenchymal transition and cell migration. J Biol Chem 275: 36803–36810 10969078 10.1074/jbc.M005912200

[embj2021108647-bib-0011] Bates RC, Mercurio AM (2003) Tumor necrosis factor‐alpha stimulates the epithelial‐to‐mesenchymal transition of human colonic organoids. Mol Biol Cell 14: 1790–1800 12802055 10.1091/mbc.E02-09-0583 PMC165077

[embj2021108647-bib-0012] Batlle E, Sancho E, Franci C, Dominguez D, Monfar M, Baulida J, Garcia De Herreros A (2000) The transcription factor snail is a repressor of E‐cadherin gene expression in epithelial tumour cells. Nat Cell Biol 2: 84–89 10655587 10.1038/35000034

[embj2021108647-bib-0013] Bergers G, Fendt SM (2021) The metabolism of cancer cells during metastasis. Nat Rev Cancer 21: 162–180 33462499 10.1038/s41568-020-00320-2 PMC8733955

[embj2021108647-bib-0014] Bhowmick NA, Chytil A, Plieth D, Gorska AE, Dumont N, Shappell S, Washington MK, Neilson EG, Moses HL (2004) TGF‐beta signaling in fibroblasts modulates the oncogenic potential of adjacent epithelia. Science 303: 848–851 14764882 10.1126/science.1090922

[embj2021108647-bib-0015] Birchmeier W, Behrens J (1994) Cadherin expression in carcinomas: role in the formation of cell junctions and the prevention of invasiveness. Biochim Biophys Acta 1198: 11–26 8199193 10.1016/0304-419x(94)90003-5

[embj2021108647-bib-0016] Blokzijl A, Dahlqvist C, Reissmann E, Falk A, Moliner A, Lendahl U, Ibanez CF (2003) Cross‐talk between the Notch and TGF‐beta signaling pathways mediated by interaction of the Notch intracellular domain with Smad3. J Cell Biol 163: 723–728 14638857 10.1083/jcb.200305112 PMC2173673

[embj2021108647-bib-0017] Bonde AK, Tischler V, Kumar S, Soltermann A, Schwendener RA (2012) Intratumoral macrophages contribute to epithelial‐mesenchymal transition in solid tumors. BMC Cancer 12: 35 22273460 10.1186/1471-2407-12-35 PMC3314544

[embj2021108647-bib-0018] Bornes L, van Scheppingen RH, Beerling E, Schelfhorst T, Ellenbroek SIJ, Seinstra D, van Rheenen J (2019) Fsp1‐mediated lineage tracing fails to detect the majority of disseminating cells undergoing EMT. Cell Rep 29: 2565–2569.e3 31775027 10.1016/j.celrep.2019.10.107 PMC6899519

[embj2021108647-bib-0019] Brabletz S, Bajdak K, Meidhof S, Burk U, Niedermann G, Firat E, Wellner U, Dimmler A, Faller G, Schubert J*et al* (2011) The ZEB1/miR‐200 feedback loop controls Notch signalling in cancer cells. EMBO J 30: 770–782 21224848 10.1038/emboj.2010.349 PMC3041948

[embj2021108647-bib-0020] Brabletz S, Brabletz T (2010) The ZEB/miR‐200 feedback loop–a motor of cellular plasticity in development and cancer? EMBO Rep 11: 670–677 20706219 10.1038/embor.2010.117 PMC2933868

[embj2021108647-bib-0021] Brabletz T, Jung A, Reu S, Porzner M, Hlubek F, Kunz‐Schughart LA, Knuechel R, Kirchner T (2001) Variable beta‐catenin expression in colorectal cancers indicates tumor progression driven by the tumor environment. Proc Natl Acad Sci U S A 98: 10356–10361 11526241 10.1073/pnas.171610498 PMC56965

[embj2021108647-bib-0022] Brabletz T, Kalluri R, Nieto MA, Weinberg RA (2018) EMT in cancer. Nat Rev Cancer 18: 128–134 29326430 10.1038/nrc.2017.118

[embj2021108647-bib-0023] Bracken CP, Gregory PA, Kolesnikoff N, Bert AG, Wang J, Shannon MF, Goodall GJ (2008) A double‐negative feedback loop between ZEB1‐SIP1 and the microRNA‐200 family regulates epithelial‐mesenchymal transition. Cancer Res 68: 7846–7854 18829540 10.1158/0008-5472.CAN-08-1942

[embj2021108647-bib-0024] Bray SJ (2016) Notch signalling in context. Nat Rev Mol Cell Biol 17: 722–735 27507209 10.1038/nrm.2016.94

[embj2021108647-bib-0025] Burk U, Schubert J, Wellner U, Schmalhofer O, Vincan E, Spaderna S, Brabletz T (2008) A reciprocal repression between ZEB1 and members of the miR‐200 family promotes EMT and invasion in cancer cells. EMBO Rep 9: 582–589 18483486 10.1038/embor.2008.74 PMC2396950

[embj2021108647-bib-0026] Byers LA, Diao L, Wang J, Saintigny P, Girard L, Peyton M, Shen Li, Fan Y, Giri U, Tumula PK*et al* (2013) An epithelial‐mesenchymal transition gene signature predicts resistance to EGFR and PI3K inhibitors and identifies Axl as a therapeutic target for overcoming EGFR inhibitor resistance. Clin Cancer Res 19: 279–290 23091115 10.1158/1078-0432.CCR-12-1558 PMC3567921

[embj2021108647-bib-0027] Calon A, Lonardo E, Berenguer‐Llergo A, Espinet E, Hernando‐Momblona X, Iglesias M, Sevillano M, Palomo‐Ponce S, Tauriello DVF, Byrom D*et al* (2015) Stromal gene expression defines poor‐prognosis subtypes in colorectal cancer. Nat Genet 47: 320–329 25706628 10.1038/ng.3225

[embj2021108647-bib-0028] Cano A, Perez‐Moreno MA, Rodrigo I, Locascio A, Blanco MJ, del Barrio MG, Portillo F, Nieto MA (2000) The transcription factor snail controls epithelial‐mesenchymal transitions by repressing E‐cadherin expression. Nat Cell Biol 2: 76–83 10655586 10.1038/35000025

[embj2021108647-bib-0029] Cao Z, Livas T, Kyprianou N (2016) Anoikis and EMT: lethal "Liaisons" during cancer progression. Crit Rev Oncog 21: 155–168 27915969 10.1615/CritRevOncog.2016016955 PMC5451151

[embj2021108647-bib-0030] Caramel J, Ligier M, Puisieux A (2018) Pleiotropic roles for ZEB1 in cancer. Cancer Res 78: 30–35 29254997 10.1158/0008-5472.CAN-17-2476

[embj2021108647-bib-0031] Chaffer CL, Brennan JP, Slavin JL, Blick T, Thompson EW, Williams ED (2006) Mesenchymal‐to‐epithelial transition facilitates bladder cancer metastasis: role of fibroblast growth factor receptor‐2. Cancer Res 66: 11271–11278 17145872 10.1158/0008-5472.CAN-06-2044

[embj2021108647-bib-0032] Che D, Zhang S, Jing Z, Shang L, Jin S, Liu F, Shen J, Li Y, Hu J, Meng Q*et al* (2017) Macrophages induce EMT to promote invasion of lung cancer cells through the IL‐6‐mediated COX‐2/PGE2/beta‐catenin signalling pathway. Mol Immunol 90: 197–210 28837884 10.1016/j.molimm.2017.06.018

[embj2021108647-bib-0033] Chen BJ, Wu JS, Tang YJ, Tang YL, Liang XH (2020) What makes leader cells arise: Intrinsic properties and support from neighboring cells. J Cell Physiol 235: 8983–8995 32572948 10.1002/jcp.29828

[embj2021108647-bib-0034] Chen L, Gibbons DL, Goswami S, Cortez MA, Ahn Y‐H, Byers LA, Zhang X, Yi X, Dwyer D, Lin W*et al* (2014) Metastasis is regulated via microRNA‐200/ZEB1 axis control of tumour cell PD‐L1 expression and intratumoral immunosuppression. Nat Commun 5: 5241 25348003 10.1038/ncomms6241 PMC4212319

[embj2021108647-bib-0035] Chen Y, Kim J, Yang S, Wang H, Wu CJ, Sugimoto H, LeBleu VS, Kalluri R (2021) Type I collagen deletion in alphaSMA(+) myofibroblasts augments immune suppression and accelerates progression of pancreatic cancer. Cancer Cell 39: 548–565.e546 33667385 10.1016/j.ccell.2021.02.007 PMC8423173

[embj2021108647-bib-0036] Cheung KJ, Padmanaban V, Silvestri V, Schipper K, Cohen JD, Fairchild AN, Gorin MA, Verdone JE, Pienta KJ, Bader JS*et al* (2016) Polyclonal breast cancer metastases arise from collective dissemination of keratin 14‐expressing tumor cell clusters. Proc Natl Acad Sci U S A 113: E854–E863 26831077 10.1073/pnas.1508541113 PMC4763783

[embj2021108647-bib-0037] Clevers H (2006) Wnt/beta‐catenin signaling in development and disease. Cell 127: 469–480 17081971 10.1016/j.cell.2006.10.018

[embj2021108647-bib-0038] Colomiere M, Ward AC, Riley C, Trenerry MK, Cameron‐Smith D, Findlay J, Ackland L, Ahmed N (2009) Cross talk of signals between EGFR and IL‐6R through JAK2/STAT3 mediate epithelial‐mesenchymal transition in ovarian carcinomas. Br J Cancer 100: 134–144 19088723 10.1038/sj.bjc.6604794 PMC2634691

[embj2021108647-bib-0039] Comaills V, Kabeche L, Morris R, Buisson R, Yu M, Madden MW, LiCausi JA, Boukhali M, Tajima K, Pan S*et al* (2016) Genomic instability is induced by persistent proliferation of cells undergoing epithelial‐to‐mesenchymal transition. Cell Rep 17: 2632–2647 27926867 10.1016/j.celrep.2016.11.022 PMC5320932

[embj2021108647-bib-0040] Comijn J, Berx G, Vermassen P, Verschueren K, van Grunsven L, Bruyneel E, Mareel M, Huylebroeck D, van Roy F (2001) The two‐handed E box binding zinc finger protein SIP1 downregulates E‐cadherin and induces invasion. Mol Cell 7: 1267–1278 11430829 10.1016/s1097-2765(01)00260-x

[embj2021108647-bib-0041] Cooke V, LeBleu V, Keskin D, Khan Z, O'Connell J, Teng Y, Duncan M, Xie L, Maeda G, Vong S*et al* (2012) Pericyte depletion results in hypoxia‐associated epithelial‐to‐mesenchymal transition and metastasis mediated by met signaling pathway. Cancer Cell 21: 66–81 22264789 10.1016/j.ccr.2011.11.024 PMC3999522

[embj2021108647-bib-0042] Cortez MA, Valdecanas D, Zhang X, Zhan Y, Bhardwaj V, Calin GA, Komaki R, Giri DK, Quini CC, Wolfe T*et al* (2014) Therapeutic delivery of miR‐200c enhances radiosensitivity in lung cancer. Mol Ther 22: 1494–1503 24791940 10.1038/mt.2014.79 PMC4435581

[embj2021108647-bib-0043] Creighton CJ, Li X, Landis M, Dixon JM, Neumeister VM, Sjolund A, Rimm DL, Wong H, Rodriguez A, Herschkowitz JI*et al* (2009) Residual breast cancers after conventional therapy display mesenchymal as well as tumor‐initiating features. Proc Natl Acad Sci U S A 106: 13820–13825 19666588 10.1073/pnas.0905718106 PMC2720409

[embj2021108647-bib-0044] de Sousa e Melo F, Kurtova AV, Harnoss JM, Kljavin N, Hoeck JD, Hung J, Anderson JE, Storm EE, Modrusan Z, Koeppen H*et al* (2017) A distinct role for Lgr5(+) stem cells in primary and metastatic colon cancer. Nature 543: 676–680 28358093 10.1038/nature21713

[embj2021108647-bib-0045] Debaugnies M, Sanchez‐Danes A, Rorive S, Raphael M, Liagre M, Parent MA, Brisebarre A, Salmon I, Blanpain C (2018) YAP and TAZ are essential for basal and squamous cell carcinoma initiation. EMBO Rep 19: e45809 10.15252/embr.201845809 PMC6030692 29875149

[embj2021108647-bib-0046] del Pozo Martin Y, Park D, Ramachandran A, Ombrato L, Calvo F, Chakravarty P, Spencer‐Dene B, Derzsi S, Hill C, Sahai E*et al* (2015) Mesenchymal cancer cell‐stroma crosstalk promotes niche activation, epithelial reversion, and metastatic colonization. Cell Rep 13: 2456–2469 26670048 10.1016/j.celrep.2015.11.025 PMC4695340

[embj2021108647-bib-0047] Derynck R, Turley SJ, Akhurst RJ (2021) TGFbeta biology in cancer progression and immunotherapy. Nat Rev Clin Oncol 18: 9–34 32710082 10.1038/s41571-020-0403-1 PMC9721352

[embj2021108647-bib-0048] Devarajan E, Song YH, Krishnappa S, Alt E (2012) Epithelial‐mesenchymal transition in breast cancer lines is mediated through PDGF‐D released by tissue‐resident stem cells. Int J Cancer 131: 1023–1031 22038895 10.1002/ijc.26493

[embj2021108647-bib-0049] Diaz‐Lopez A, Moreno‐Bueno G, Cano A (2014) Role of microRNA in epithelial to mesenchymal transition and metastasis and clinical perspectives. Cancer Manag Res 6: 205–216 24812525 10.2147/CMAR.S38156 PMC4008290

[embj2021108647-bib-0050] Diepenbruck M, Waldmeier L, Ivanek R, Berninger P, Arnold P, van Nimwegen E, Christofori G (2014) Tead2 expression levels control the subcellular distribution of Yap and Taz, zyxin expression and epithelial‐mesenchymal transition. J Cell Sci 127: 1523–1536 24554433 10.1242/jcs.139865

[embj2021108647-bib-0051] Dongre A, Rashidian M, Reinhardt F, Bagnato A, Keckesova Z, Ploegh HL, Weinberg RA (2017) Epithelial‐to‐mesenchymal transition contributes to immunosuppression in breast carcinomas. Cancer Res 77: 3982–3989 28428275 10.1158/0008-5472.CAN-16-3292 PMC5541771

[embj2021108647-bib-0052] Dongre A, Weinberg RA (2019) New insights into the mechanisms of epithelial‐mesenchymal transition and implications for cancer. Nat Rev Mol Cell Biol 20: 69–84 30459476 10.1038/s41580-018-0080-4

[embj2021108647-bib-0053] Dudas J, Ladanyi A, Ingruber J, Steinbichler TB, Riechelmann H (2020) Epithelial to mesenchymal transition: a mechanism that fuels cancer radio/chemoresistance. Cells 9: 428 10.3390/cells9020428 PMC7072371 32059478

[embj2021108647-bib-0054] Eckert MA, Lwin TM, Chang AT, Kim J, Danis E, Ohno‐Machado L, Yang J (2011) Twist1‐induced invadopodia formation promotes tumor metastasis. Cancer Cell 19: 372–386 21397860 10.1016/j.ccr.2011.01.036 PMC3072410

[embj2021108647-bib-0055] Eger A, Aigner K, Sonderegger S, Dampier B, Oehler S, Schreiber M, Berx G, Cano A, Beug H, Foisner R (2005) DeltaEF1 is a transcriptional repressor of E‐cadherin and regulates epithelial plasticity in breast cancer cells. Oncogene 24: 2375–2385 15674322 10.1038/sj.onc.1208429

[embj2021108647-bib-0056] Elyada E, Bolisetty M, Laise P, Flynn WF, Courtois ET, Burkhart RA, Teinor JA, Belleau P, Biffi G, Lucito MS*et al* (2019) Cross‐species single‐cell analysis of pancreatic ductal adenocarcinoma reveals antigen‐presenting cancer‐associated fibroblasts. Cancer Discov 9: 1102–1123 31197017 10.1158/2159-8290.CD-19-0094 PMC6727976

[embj2021108647-bib-0057] Escrivà M, Peiró S, Herranz N, Villagrasa P, Dave N, Montserrat‐Sentís B, Murray SA, Francí C, Gridley T, Virtanen I*et al* (2008) Repression of PTEN phosphatase by Snail1 transcriptional factor during gamma radiation‐induced apoptosis. Mol Cell Biol 28: 1528–1540 18172008 10.1128/MCB.02061-07 PMC2258777

[embj2021108647-bib-0058] Esposito M, Mondal N, Greco TM, Wei Y, Spadazzi C, Lin S‐C, Zheng H, Cheung C, Magnani JL, Lin S‐H*et al* (2019) Bone vascular niche E‐selectin induces mesenchymal‐epithelial transition and Wnt activation in cancer cells to promote bone metastasis. Nat Cell Biol 21: 627–639 30988423 10.1038/s41556-019-0309-2 PMC6556210

[embj2021108647-bib-0059] Fan Q‐M, Jing Y‐Y, Yu G‐F, Kou X‐R, Ye F, Gao Lu, Li R, Zhao Q‐D, Yang Y, Lu Z‐H*et al* (2014) Tumor‐associated macrophages promote cancer stem cell‐like properties via transforming growth factor‐beta1‐induced epithelial‐mesenchymal transition in hepatocellular carcinoma. Cancer Lett 352: 160–168 24892648 10.1016/j.canlet.2014.05.008

[embj2021108647-bib-0060] Farmer P, Bonnefoi H, Anderle P, Cameron D, Wirapati P, Becette V, André S, Piccart M, Campone M, Brain E*et al* (2009) A stroma‐related gene signature predicts resistance to neoadjuvant chemotherapy in breast cancer. Nat Med 15: 68–74 19122658 10.1038/nm.1908

[embj2021108647-bib-0061] Fearon ER, Vogelstein B (1990) A genetic model for colorectal tumorigenesis. Cell 61: 759–767 2188735 10.1016/0092-8674(90)90186-i

[embj2021108647-bib-0062] Feldker N, Ferrazzi F, Schuhwerk H, Widholz SA, Guenther K, Frisch I, Jakob K, Kleemann J, Riegel D, Bonisch U*et al* (2020) Genome‐wide cooperation of EMT transcription factor ZEB1 with YAP and AP‐1 in breast cancer. EMBO J 39: e103209 32692442 10.15252/embj.2019103209 PMC7459422

[embj2021108647-bib-0063] Fifis T, Nguyen L, Malcontenti‐Wilson C, Chan LS, Nunes Costa PL, Daruwalla J, Nikfarjam M, Muralidharan V, Waltham M, Thompson EW*et al* (2013) Treatment with the vascular disruptive agent OXi4503 induces an immediate and widespread epithelial to mesenchymal transition in the surviving tumor. Cancer Med 2: 595–610 24403226 10.1002/cam4.109 PMC3892792

[embj2021108647-bib-0064] Fischer KR, Durrans A, Lee S, Sheng J, Li F, Wong STC, Choi H, El Rayes T, Ryu S, Troeger J*et al* (2015) Epithelial‐to‐mesenchymal transition is not required for lung metastasis but contributes to chemoresistance. Nature 527: 472–476 26560033 10.1038/nature15748 PMC4662610

[embj2021108647-bib-0065] Friedl P, Locker J, Sahai E, Segall JE (2012) Classifying collective cancer cell invasion. Nat Cell Biol 14: 777–783 22854810 10.1038/ncb2548

[embj2021108647-bib-0066] Fumagalli A, Oost KC, Kester L, Morgner J, Bornes L, Bruens L, Spaargaren L, Azkanaz M, Schelfhorst T, Beerling E*et al* (2020) Plasticity of Lgr5‐negative cancer cells drives metastasis in colorectal cancer. Cell Stem Cell 26: 569–578.e7 32169167 10.1016/j.stem.2020.02.008 PMC7118369

[embj2021108647-bib-0067] Gabrilovich DI (2017) Myeloid‐derived suppressor cells. Cancer Immunol Res 5: 3–8 28052991 10.1158/2326-6066.CIR-16-0297 PMC5426480

[embj2021108647-bib-0068] Gabrilovich DI, Nagaraj S (2009) Myeloid‐derived suppressor cells as regulators of the immune system. Nat Rev Immunol 9: 162–174 19197294 10.1038/nri2506 PMC2828349

[embj2021108647-bib-0069] Gauger KJ, Chenausky KL, Murray ME, Schneider SS (2011) SFRP1 reduction results in an increased sensitivity to TGF‐beta signaling. BMC Cancer 11: 59 21303533 10.1186/1471-2407-11-59 PMC3041779

[embj2021108647-bib-0070] Geng Y, Fan J, Chen L, Zhang C, Qu C, Qian L, Chen K, Meng Z, Chen Z, Wang P (2021) A notch‐dependent inflammatory feedback circuit between macrophages and cancer cells regulates pancreatic cancer metastasis. Cancer Res 81: 64–76 33172931 10.1158/0008-5472.CAN-20-0256

[embj2021108647-bib-0071] Georgakopoulos‐Soares I, Chartoumpekis DV, Kyriazopoulou V, Zaravinos A (2020) EMT factors and metabolic pathways in cancer. Front Oncol 10: 499 32318352 10.3389/fonc.2020.00499 PMC7154126

[embj2021108647-bib-0072] Goebel L, Grage‐Griebenow E, Gorys A, Helm O, Genrich G, Lenk L, Wesch D, Ungefroren H, Freitag‐Wolf S, Sipos B*et al* (2015) CD4(+) T cells potently induce epithelial‐mesenchymal‐transition in premalignant and malignant pancreatic ductal epithelial cells‐novel implications of CD4(+) T cells in pancreatic cancer development. Oncoimmunology 4: e1000083 26137395 10.1080/2162402X.2014.1000083 PMC4485733

[embj2021108647-bib-0073] Gonzalez H, Hagerling C, Werb Z (2018) Roles of the immune system in cancer: from tumor initiation to metastatic progression. Genes Dev 32: 1267–1284 30275043 10.1101/gad.314617.118 PMC6169832

[embj2021108647-bib-0074] Goulet CR, Champagne A, Bernard G, Vandal D, Chabaud S, Pouliot F, Bolduc S (2019) Cancer‐associated fibroblasts induce epithelial‐mesenchymal transition of bladder cancer cells through paracrine IL‐6 signalling. BMC Cancer 19: 137 30744595 10.1186/s12885-019-5353-6 PMC6371428

[embj2021108647-bib-0075] Graham TR, Zhau HE, Odero‐Marah VA, Osunkoya AO, Kimbro KS, Tighiouart M, Liu T, Simons JW, O'Regan RM (2008) Insulin‐like growth factor‐I‐dependent up‐regulation of ZEB1 drives epithelial‐to‐mesenchymal transition in human prostate cancer cells. Cancer Res 68: 2479–2488 18381457 10.1158/0008-5472.CAN-07-2559

[embj2021108647-bib-0076] Gross KM, Zhou W, Breindel JL, Ouyang J, Jin DX, Sokol ES, Gupta PB, Huber K, Zou L, Kuperwasser C (2019) Loss of slug compromises DNA damage repair and accelerates stem cell aging in mammary epithelium. Cell Rep 28: 394–407.e6 31291576 10.1016/j.celrep.2019.06.043

[embj2021108647-bib-0077] Grotegut S, von Schweinitz D, Christofori G, Lehembre F (2006) Hepatocyte growth factor induces cell scattering through MAPK/Egr‐1‐mediated upregulation of Snail. EMBO J 25: 3534–3545 16858414 10.1038/sj.emboj.7601213 PMC1538570

[embj2021108647-bib-0078] Gubelmann C, Schwalie PC, Raghav SK, Röder E, Delessa T, Kiehlmann E, Waszak SM, Corsinotti A, Udin G, Holcombe W*et al* (2014) Identification of the transcription factor ZEB1 as a central component of the adipogenic gene regulatory network. Elife 3: e03346 25163748 10.7554/eLife.03346 PMC4359378

[embj2021108647-bib-0079] Gudey SK, Sundar R, Heldin CH, Bergh A, Landstrom M (2017) Pro‐invasive properties of Snail1 are regulated by sumoylation in response to TGFbeta stimulation in cancer. Oncotarget 8: 97703–97726 29228645 10.18632/oncotarget.20097 PMC5716685

[embj2021108647-bib-0080] Guo W, Keckesova Z, Donaher J, Shibue T, Tischler V, Reinhardt F, Itzkovitz S, Noske A, Zürrer‐Härdi U, Bell G*et al* (2012) Slug and Sox9 cooperatively determine the mammary stem cell state. Cell 148: 1015–1028 22385965 10.1016/j.cell.2012.02.008 PMC3305806

[embj2021108647-bib-0081] Gupta PB, Onder TT, Jiang G, Tao K, Kuperwasser C, Weinberg RA, Lander ES (2009) Identification of selective inhibitors of cancer stem cells by high‐throughput screening. Cell 138: 645–659 19682730 10.1016/j.cell.2009.06.034 PMC4892125

[embj2021108647-bib-0082] Halazonetis TD, Gorgoulis VG, Bartek J (2008) An oncogene‐induced DNA damage model for cancer development. Science 319: 1352–1355 18323444 10.1126/science.1140735

[embj2021108647-bib-0083] Hanahan D, Weinberg RA (2011) Hallmarks of cancer: the next generation. Cell 144: 646–674 21376230 10.1016/j.cell.2011.02.013

[embj2021108647-bib-0084] Hao Y, Baker D, Ten Dijke P (2019) TGF‐beta‐mediated epithelial‐mesenchymal transition and cancer metastasis. Int J Mol Sci 20: 2767 10.3390/ijms20112767 PMC6600375 31195692

[embj2021108647-bib-0085] Hapke RY, Haake SM (2020) Hypoxia‐induced epithelial to mesenchymal transition in cancer. Cancer Lett 487: 10–20 32470488 10.1016/j.canlet.2020.05.012 PMC7336507

[embj2021108647-bib-0086] Hara J, Miyata H, Yamasaki M, Sugimura K, Takahashi T, Kurokawa Y, Nakajima K, Takiguchi S, Mori M, Doki Y (2014) Mesenchymal phenotype after chemotherapy is associated with chemoresistance and poor clinical outcome in esophageal cancer. Oncol Rep 31: 589–596 24297447 10.3892/or.2013.2876

[embj2021108647-bib-0087] Hay ED (1995) An overview of epithelio‐mesenchymal transformation. Acta Anat (Basel) 154: 8–20 8714286 10.1159/000147748

[embj2021108647-bib-0088] He X, Yan B, Liu S, Jia J, Lai W, Xin X, Tang C‐e, Luo D, Tan T, Jiang Y*et al* (2016) Chromatin remodeling factor LSH drives cancer progression by suppressing the activity of fumarate hydratase. Cancer Res 76: 5743–5755 27302170 10.1158/0008-5472.CAN-16-0268 PMC7821962

[embj2021108647-bib-0089] Ho MY, Tang SJ, Chuang MJ, Cha TL, Li JY, Sun GH, Sun KH (2012) TNF‐alpha induces epithelial‐mesenchymal transition of renal cell carcinoma cells via a GSK3beta‐dependent mechanism. Mol Cancer Res 10: 1109–1119 22707636 10.1158/1541-7786.MCR-12-0160

[embj2021108647-bib-0090] Hoot KE, Lighthall J, Han G, Lu SL, Li A, Ju W, Kulesz‐Martin M, Bottinger E, Wang XJ (2008) Keratinocyte‐specific Smad2 ablation results in increased epithelial‐mesenchymal transition during skin cancer formation and progression. J Clin Invest 118: 2722–2732 18618014 10.1172/JCI33713 PMC2447925

[embj2021108647-bib-0091] Hua W, Ten Dijke P, Kostidis S, Giera M, Hornsveld M (2020) TGFbeta‐induced metabolic reprogramming during epithelial‐to‐mesenchymal transition in cancer. Cell Mol Life Sci 77: 2103–2123 31822964 10.1007/s00018-019-03398-6 PMC7256023

[embj2021108647-bib-0092] Huang CH, Yang WH, Chang SY, Tai SK, Tzeng CH, Kao JY, Wu KJ, Yang MH (2009) Regulation of membrane‐type 4 matrix metalloproteinase by SLUG contributes to hypoxia‐mediated metastasis. Neoplasia 11: 1371–1382 20019845 10.1593/neo.91326 PMC2794518

[embj2021108647-bib-0093] Huang RY, Guilford P, Thiery JP (2012) Early events in cell adhesion and polarity during epithelial‐mesenchymal transition. J Cell Sci 125: 4417–4422 23165231 10.1242/jcs.099697

[embj2021108647-bib-0094] Hugo W, Zaretsky JM, Sun Lu, Song C, Moreno BH, Hu‐Lieskovan S, Berent‐Maoz B, Pang J, Chmielowski B, Cherry G*et al* (2016) Genomic and transcriptomic features of response to anti‐PD‐1 therapy in metastatic melanoma. Cell 165: 35–44 26997480 10.1016/j.cell.2016.02.065 PMC4808437

[embj2021108647-bib-0095] Ieda T, Tazawa H, Okabayashi H, Yano S, Shigeyasu K, Kuroda S, Ohara T, Noma K, Kishimoto H, Nishizaki M*et al* (2019) Visualization of epithelial‐mesenchymal transition in an inflammatory microenvironment‐colorectal cancer network. Sci Rep 9: 16378 31705021 10.1038/s41598-019-52816-z PMC6841984

[embj2021108647-bib-0096] Ishay‐Ronen D, Diepenbruck M, Kalathur RKR, Sugiyama N, Tiede S, Ivanek R, Bantug G, Morini MF, Wang J, Hess C*et al* (2019) Gain fat‐lose metastasis: converting invasive breast cancer cells into adipocytes inhibits cancer metastasis. Cancer Cell 35: 17–32.e6 30645973 10.1016/j.ccell.2018.12.002

[embj2021108647-bib-0097] Jiang YP, Tang YL, Wang SS, Wu JS, Zhang M, Pang X, Wu JB, Chen Y, Tang YJ, Liang XH (2020) PRRX1‐induced epithelial‐to‐mesenchymal transition in salivary adenoid cystic carcinoma activates the metabolic reprogramming of free fatty acids to promote invasion and metastasis. Cell Prolif 53: e12705 31657086 10.1111/cpr.12705 PMC6985691

[embj2021108647-bib-0098] Jiang Y, Zhan H (2020) Communication between EMT and PD‐L1 signaling: new insights into tumor immune evasion. Cancer Lett 468: 72–81 31605776 10.1016/j.canlet.2019.10.013

[embj2021108647-bib-0099] Jin MZ, Jin WL (2020) The updated landscape of tumor microenvironment and drug repurposing. Signal Transduct Target Ther 5: 166 32843638 10.1038/s41392-020-00280-x PMC7447642

[embj2021108647-bib-0100] Joffroy CM, Buck MB, Stope MB, Popp SL, Pfizenmaier K, Knabbe C (2010) Antiestrogens induce transforming growth factor beta‐mediated immunosuppression in breast cancer. Cancer Res 70: 1314–1322 20145137 10.1158/0008-5472.CAN-09-3292

[embj2021108647-bib-0101] Jonckheere S, Adams J, De Groote D, Campbell K, Berx G, Goossens S (2021) Epithelial‐Mesenchymal Transition (EMT) as a therapeutic target. Cells Tissues Organs 1–26. 10.1159/000512218 33401271

[embj2021108647-bib-0102] Joyce JA, Pollard JW (2009) Microenvironmental regulation of metastasis. Nat Rev Cancer 9: 239–252 19279573 10.1038/nrc2618 PMC3251309

[embj2021108647-bib-0103] Jung AR, Jung CH, Noh JK, Lee YC, Eun YG (2020) Epithelial‐mesenchymal transition gene signature is associated with prognosis and tumor microenvironment in head and neck squamous cell carcinoma. Sci Rep 10: 3652 32107458 10.1038/s41598-020-60707-x PMC7046610

[embj2021108647-bib-0104] Kajita M, McClinic KN, Wade PA (2004) Aberrant expression of the transcription factors snail and slug alters the response to genotoxic stress. Mol Cell Biol 24: 7559–7566 15314165 10.1128/MCB.24.17.7559-7566.2004 PMC506998

[embj2021108647-bib-0105] Kang H, Kim H, Lee S, Youn H, Youn B (2019) Role of metabolic reprogramming in Epithelial(‐)Mesenchymal Transition (EMT). Int J Mol Sci 20: 2042 10.3390/ijms20082042 PMC6514888 31027222

[embj2021108647-bib-0106] Kardos J, Chai S, Mose LE, Selitsky SR, Krishnan B, Saito R, Iglesia MD, Milowsky MI, Parker JS, Kim WY*et al* (2016) Claudin‐low bladder tumors are immune infiltrated and actively immune suppressed. JCI Insight 1: e85902 27699256 10.1172/jci.insight.85902 PMC5033914

[embj2021108647-bib-0107] Katsura A, Tamura Y, Hokari S, Harada M, Morikawa M, Sakurai T, Takahashi K, Mizutani A, Nishida J, Yokoyama Y*et al* (2017) ZEB1‐regulated inflammatory phenotype in breast cancer cells. Mol Oncol 11: 1241–1262 28618162 10.1002/1878-0261.12098 PMC5579340

[embj2021108647-bib-0108] Kawamoto A, Yokoe T, Tanaka K, Saigusa S, Toiyama Y, Yasuda H, Inoue Y, Miki C, Kusunoki M (2012) Radiation induces epithelial‐mesenchymal transition in colorectal cancer cells. Oncol Rep 27: 51–57 21971767 10.3892/or.2011.1485

[embj2021108647-bib-0109] Khot M, Sreekumar D, Jahagirdar S, Kulkarni A, Hari K, Faseela EE, Sabarinathan R, Jolly MK, Sengupta K (2020) Twist1 induces chromosomal instability (CIN) in colorectal cancer cells. Hum Mol Genet 29: 1673–1688 32337580 10.1093/hmg/ddaa076 PMC7322571

[embj2021108647-bib-0110] Kim J, Kong J, Chang H, Kim H, Kim A (2016) EGF induces epithelial‐mesenchymal transition through phospho‐Smad2/3‐Snail signaling pathway in breast cancer cells. Oncotarget 7: 85021–85032 27829223 10.18632/oncotarget.13116 PMC5356716

[embj2021108647-bib-0111] Kim T, Veronese A, Pichiorri F, Lee TJ, Jeon Y‐J, Volinia S, Pineau P, Marchio A, Palatini J, Suh S‐S*et al* (2011) p53 regulates epithelial‐mesenchymal transition through microRNAs targeting ZEB1 and ZEB2. J Exp Med 208: 875–883 21518799 10.1084/jem.20110235 PMC3092351

[embj2021108647-bib-0112] Kmieciak M, Knutson KL, Dumur CI, Manjili MH (2007) HER‐2/neu antigen loss and relapse of mammary carcinoma are actively induced by T cell‐mediated anti‐tumor immune responses. Eur J Immunol 37: 675–685 17304628 10.1002/eji.200636639 PMC3732067

[embj2021108647-bib-0113] Korpal M, Ell BJ, Buffa FM, Ibrahim T, Blanco MA, Celià‐Terrassa T, Mercatali L, Khan Z, Goodarzi H, Hua Y*et al* (2011) Direct targeting of Sec23a by miR‐200s influences cancer cell secretome and promotes metastatic colonization. Nat Med 17: 1101–1108 21822286 10.1038/nm.2401 PMC3169707

[embj2021108647-bib-0114] Kothari AN, Mi Z, Zapf M, Kuo PC (2014) Novel clinical therapeutics targeting the epithelial to mesenchymal transition. Clin Transl Med 3: 35 25343018 10.1186/s40169-014-0035-0 PMC4198571

[embj2021108647-bib-0115] Krebs AM, Mitschke J, Lasierra Losada M, Schmalhofer O, Boerries M, Busch H, Boettcher M, Mougiakakos D, Reichardt W, Bronsert P*et al* (2017) The EMT‐activator Zeb1 is a key factor for cell plasticity and promotes metastasis in pancreatic cancer. Nat Cell Biol 19: 518–529 28414315 10.1038/ncb3513

[embj2021108647-bib-0116] Kudo‐Saito C, Ozaki Y, Imazeki H, Hayashi H, Masuda J, Ozawa H, Ogiwara Y (2021) Targeting oncoimmune drivers of cancer metastasis. Cancers (Basel) 13: 554 33535613 10.3390/cancers13030554 PMC7867187

[embj2021108647-bib-0117] Kudo‐Saito C, Shirako H, Takeuchi T, Kawakami Y (2009) Cancer metastasis is accelerated through immunosuppression during Snail‐induced EMT of cancer cells. Cancer Cell 15: 195–206 19249678 10.1016/j.ccr.2009.01.023

[embj2021108647-bib-0118] Kumar V, Donthireddy L, Marvel D, Condamine T, Wang F, Lavilla‐Alonso S, Hashimoto A, Vonteddu P, Behera R, Goins MA*et al* (2017) Cancer‐associated fibroblasts neutralize the anti‐tumor effect of CSF1 receptor blockade by inducing PMN‐MDSC infiltration of tumors. Cancer Cell 32: 654–668.e5 29136508 10.1016/j.ccell.2017.10.005 PMC5827952

[embj2021108647-bib-0119] Kurrey NK, Jalgaonkar SP, Joglekar AV, Ghanate AD, Chaskar PD, Doiphode RY, Bapat SA (2009) Snail and slug mediate radioresistance and chemoresistance by antagonizing p53‐mediated apoptosis and acquiring a stem‐like phenotype in ovarian cancer cells. Stem Cells 27: 2059–2068 19544473 10.1002/stem.154

[embj2021108647-bib-0120] Lamar JM, Stern P, Liu H, Schindler JW, Jiang ZG, Hynes RO (2012) The Hippo pathway target, YAP, promotes metastasis through its TEAD‐interaction domain. Proc Natl Acad Sci U S A 109: E2441–E2450 22891335 10.1073/pnas.1212021109 PMC3443162

[embj2021108647-bib-0121] Lambrechts D, Wauters E, Boeckx B, Aibar S, Nittner D, Burton O, Bassez A, Decaluwé H, Pircher A, Van den Eynde K*et al* (2018) Phenotype molding of stromal cells in the lung tumor microenvironment. Nat Med 24: 1277–1289 29988129 10.1038/s41591-018-0096-5

[embj2021108647-bib-0122] Lamouille S, Connolly E, Smyth JW, Akhurst RJ, Derynck R (2012) TGF‐beta‐induced activation of mTOR complex 2 drives epithelial‐mesenchymal transition and cell invasion. J Cell Sci 125: 1259–1273 22399812 10.1242/jcs.095299 PMC3324583

[embj2021108647-bib-0123] Lamouille S, Derynck R (2007) Cell size and invasion in TGF‐beta‐induced epithelial to mesenchymal transition is regulated by activation of the mTOR pathway. J Cell Biol 178: 437–451 17646396 10.1083/jcb.200611146 PMC2064840

[embj2021108647-bib-0124] Lamouille S, Xu J, Derynck R (2014) Molecular mechanisms of epithelial‐mesenchymal transition. Nat Rev Mol Cell Biol 15: 178–196 24556840 10.1038/nrm3758 PMC4240281

[embj2021108647-bib-0125] Larsen JE, Nathan V, Osborne JK, Farrow RK, Deb D, Sullivan JP, Dospoy PD, Augustyn A, Hight SK, Sato M*et al* (2016) ZEB1 drives epithelial‐to‐mesenchymal transition in lung cancer. J Clin Invest 126: 3219–3235 27500490 10.1172/JCI76725 PMC5004933

[embj2021108647-bib-0126] LeBleu VS, Kalluri R (2018) A peek into cancer‐associated fibroblasts: origins, functions and translational impact. Dis Model Mech 11: dmm029447 29686035 10.1242/dmm.029447 PMC5963854

[embj2021108647-bib-0127] Lee CC, Lin JC, Hwang WL, Kuo YJ, Chen HK, Tai SK, Lin CC, Yang MH (2018) Macrophage‐secreted interleukin‐35 regulates cancer cell plasticity to facilitate metastatic colonization. Nat Commun 9: 3763 30218063 10.1038/s41467-018-06268-0 PMC6138674

[embj2021108647-bib-0128] Lehmann W, Mossmann D, Kleemann J, Mock K, Meisinger C, Brummer T, Herr R, Brabletz S, Stemmler MP, Brabletz T (2016) ZEB1 turns into a transcriptional activator by interacting with YAP1 in aggressive cancer types. Nat Commun 7: 10498 26876920 10.1038/ncomms10498 PMC4756710

[embj2021108647-bib-0129] Li S, Cong X, Gao H, Lan X, Li Z, Wang W, Song S, Wang Y, Li C, Zhang H*et al* (2019) Tumor‐associated neutrophils induce EMT by IL‐17a to promote migration and invasion in gastric cancer cells. J Exp Clin Cancer Res 38: 6 30616627 10.1186/s13046-018-1003-0 PMC6323742

[embj2021108647-bib-0130] Li Y, Wang L, Pappan L, Galliher‐Beckley A, Shi J (2012) IL‐1beta promotes stemness and invasiveness of colon cancer cells through Zeb1 activation. Mol Cancer 11: 87 23174018 10.1186/1476-4598-11-87 PMC3532073

[embj2021108647-bib-0131] Li Z, Wang Y, Zhu Y, Yuan C, Wang D, Zhang W, Qi B, Qiu J, Song X, Ye J*et al* (2015) The Hippo transducer TAZ promotes epithelial to mesenchymal transition and cancer stem cell maintenance in oral cancer. Mol Oncol 9: 1091–1105 25704916 10.1016/j.molonc.2015.01.007 PMC5528756

[embj2021108647-bib-0132] Lin JC, Tsai JT, Chao TY, Ma HI, Liu WH (2018) The STAT3/slug axis enhances radiation‐induced tumor invasion and cancer stem‐like properties in radioresistant glioblastoma. Cancers 10: 512 10.3390/cancers10120512 PMC6315497 30551687

[embj2021108647-bib-0133] Liu J, Ruan B, You N, Huang Q, Liu W, Dang Z, Xu W, Zhou T, Ji R, Cao Y*et al* (2013) Downregulation of miR‐200a induces EMT phenotypes and CSC‐like signatures through targeting the beta‐catenin pathway in hepatic oval cells. PLoS One 8: e79409 24260215 10.1371/journal.pone.0079409 PMC3829824

[embj2021108647-bib-0134] Liu L, Chen X, Wang Y, Qu Z, Lu Q, Zhao J, Yan X, Zhang H, Zhou Y (2014a) Notch3 is important for TGF‐beta‐induced epithelial‐mesenchymal transition in non‐small cell lung cancer bone metastasis by regulating ZEB‐1. Cancer Gene Ther 21: 364–372 25080992 10.1038/cgt.2014.39

[embj2021108647-bib-0135] Liu S, Ren J, Ten Dijke P (2021) Targeting TGFbeta signal transduction for cancer therapy. Signal Transduct Target Ther 6: 8 33414388 10.1038/s41392-020-00436-9 PMC7791126

[embj2021108647-bib-0136] Liu Y, Lu X, Huang Li, Wang W, Jiang G, Dean KC, Clem B, Telang S, Jenson AB, Cuatrecasas M*et al* (2014b) Different thresholds of ZEB1 are required for Ras‐mediated tumour initiation and metastasis. Nat Commun 5: 5660 25434817 10.1038/ncomms6660

[embj2021108647-bib-0137] Liu Z, Tu K, Wang Y, Yao B, Li Q, Wang L, Dou C, Liu Q, Zheng X (2017) Hypoxia accelerates aggressiveness of hepatocellular carcinoma cells involving oxidative stress, epithelial‐mesenchymal transition and non‐canonical hedgehog signaling. Cell Physiol Biochem 44: 1856–1868 29237157 10.1159/000485821

[embj2021108647-bib-0138] Lo HW, Hsu SC, Xia W, Cao X, Shih JY, Wei Y, Abbruzzese JL, Hortobagyi GN, Hung MC (2007) Epidermal growth factor receptor cooperates with signal transducer and activator of transcription 3 to induce epithelial‐mesenchymal transition in cancer cells via up‐regulation of TWIST gene expression. Cancer Res 67: 9066–9076 17909010 10.1158/0008-5472.CAN-07-0575 PMC2570961

[embj2021108647-bib-0139] Lu Z, Ghosh S, Wang Z, Hunter T (2003) Downregulation of caveolin‐1 function by EGF leads to the loss of E‐cadherin, increased transcriptional activity of beta‐catenin, and enhanced tumor cell invasion. Cancer Cell 4: 499–515 14706341 10.1016/s1535-6108(03)00304-0

[embj2021108647-bib-0140] Lyons JG, Patel V, Roue NC, Fok SY, Soon LL, Halliday GM, Gutkind JS (2008) Snail up‐regulates proinflammatory mediators and inhibits differentiation in oral keratinocytes. Cancer Res 68: 4525–4530 18559496 10.1158/1078-0432.CCR-07-6735 PMC2631428

[embj2021108647-bib-0141] Ma X, Wang M, Yin T, Zhao Y, Wei X (2019) Myeloid‐derived suppressor cells promote metastasis in breast cancer after the stress of operative removal of the primary cancer. Front Oncol 9: 855 31552179 10.3389/fonc.2019.00855 PMC6746963

[embj2021108647-bib-0142] Maehara O, Suda G, Natsuizaka M, Ohnishi S, Komatsu Y, Sato F, Nakai M, Sho T, Morikawa K, Ogawa K*et al* (2017) Fibroblast growth factor‐2‐mediated FGFR/Erk signaling supports maintenance of cancer stem‐like cells in esophageal squamous cell carcinoma. Carcinogenesis 38: 1073–1083 28927233 10.1093/carcin/bgx095 PMC5862278

[embj2021108647-bib-0143] Makrodouli E, Oikonomou E, Koc M, Andera L, Sasazuki T, Shirasawa S, Pintzas A (2011) BRAF and RAS oncogenes regulate Rho GTPase pathways to mediate migration and invasion properties in human colon cancer cells: a comparative study. Mol Cancer 10: 118 21943101 10.1186/1476-4598-10-118 PMC3189908

[embj2021108647-bib-0144] Mani SA, Guo W, Liao M‐J, Eaton EN, Ayyanan A, Zhou AY, Brooks M, Reinhard F, Zhang CC, Shipitsin M*et al* (2008) The epithelial‐mesenchymal transition generates cells with properties of stem cells. Cell 133: 704–715 18485877 10.1016/j.cell.2008.03.027 PMC2728032

[embj2021108647-bib-0145] Mao Z, Zhang J, Shi Y, Li W, Shi H, Ji R, Mao F, Qian H, Xu W, Zhang X (2020) CXCL5 promotes gastric cancer metastasis by inducing epithelial‐mesenchymal transition and activating neutrophils. Oncogenesis 9: 63 32632106 10.1038/s41389-020-00249-z PMC7338464

[embj2021108647-bib-0146] Mareel M, Vleminckx K, Vermeulen S, Bracke M, Van Roy F (1992) E‐cadherin expression: a counterbalance for cancer cell invasion. Bull Cancer 79: 347–355 1421692

[embj2021108647-bib-0147] Martin‐Belmonte F, Perez‐Moreno M (2011) Epithelial cell polarity, stem cells and cancer. Nat Rev Cancer 12: 23–38 22169974 10.1038/nrc3169

[embj2021108647-bib-0148] Masin M, Vazquez J, Rossi S, Groeneveld S, Samson N, Schwalie PC, Deplancke B, Frawley LE, Gouttenoire J, Moradpour D*et al* (2014) GLUT3 is induced during epithelial‐mesenchymal transition and promotes tumor cell proliferation in non‐small cell lung cancer. Cancer Metab 2: 11 25097756 10.1186/2049-3002-2-11 PMC4122054

[embj2021108647-bib-0149] Massague J (2012) TGFbeta signalling in context. Nat Rev Mol Cell Biol 13: 616–630 22992590 10.1038/nrm3434 PMC4027049

[embj2021108647-bib-0150] Masucci MT, Minopoli M, Carriero MV (2019) Tumor associated neutrophils. Their role in tumorigenesis, metastasis, prognosis and therapy. Front Oncol 9: 1146 31799175 10.3389/fonc.2019.01146 PMC6874146

[embj2021108647-bib-0151] Matise LA, Palmer TD, Ashby WJ, Nashabi A, Chytil A, Aakre M, Pickup MW, Gorska AE, Zijlstra A, Moses HL (2012) Lack of transforming growth factor‐beta signaling promotes collective cancer cell invasion through tumor‐stromal crosstalk. Breast Cancer Res 14: R98 22748014 10.1186/bcr3217 PMC3680921

[embj2021108647-bib-0152] McGranahan N, Swanton C (2017) Clonal heterogeneity and tumor evolution: past, present, and the future. Cell 168: 613–628 28187284 10.1016/j.cell.2017.01.018

[embj2021108647-bib-0153] Meidhof S, Brabletz S, Lehmann W, Preca BT, Mock K, Ruh M, Schuler J, Berthold M, Weber A, Burk U*et al* (2015) ZEB1‐associated drug resistance in cancer cells is reversed by the class I HDAC inhibitor mocetinostat. EMBO Mol Med 7: 831–847 25872941 10.15252/emmm.201404396 PMC4459821

[embj2021108647-bib-0154] Miao JW, Liu LJ, Huang J (2014) Interleukin‐6‐induced epithelial‐mesenchymal transition through signal transducer and activator of transcription 3 in human cervical carcinoma. Int J Oncol 45: 165–176 24806843 10.3892/ijo.2014.2422

[embj2021108647-bib-0155] Miyoshi A, Kitajima Y, Sumi K, Sato K, Hagiwara A, Koga Y, Miyazaki K (2004) Snail and SIP1 increase cancer invasion by upregulating MMP family in hepatocellular carcinoma cells. Br J Cancer 90: 1265–1273 15026811 10.1038/sj.bjc.6601685 PMC2409652

[embj2021108647-bib-0156] Miyoshi A, Kitajima Y, Kido S, Shimonishi T, Matsuyama S, Kitahara K, Miyazaki K (2005) Snail accelerates cancer invasion by upregulating MMP expression and is associated with poor prognosis of hepatocellular carcinoma. Br J Cancer 92: 252–258 15668718 10.1038/sj.bjc.6602266 PMC2361838

[embj2021108647-bib-0157] Morel A‐P, Ginestier C, Pommier RM, Cabaud O, Ruiz E, Wicinski J, Devouassoux‐Shisheboran M, Combaret V, Finetti P, Chassot C*et al* (2017) A stemness‐related ZEB1‐MSRB3 axis governs cellular pliancy and breast cancer genome stability. Nat Med 23: 568–578 28394329 10.1038/nm.4323

[embj2021108647-bib-0158] Morel A‐P, Hinkal GW, Thomas C, Fauvet F, Courtois‐Cox S, Wierinckx A, Devouassoux‐Shisheboran M, Treilleux I, Tissier A, Gras B*et al* (2012) EMT inducers catalyze malignant transformation of mammary epithelial cells and drive tumorigenesis towards claudin‐low tumors in transgenic mice. PLoS Genet 8: e1002723 22654675 10.1371/journal.pgen.1002723 PMC3359981

[embj2021108647-bib-0159] Morel AP, Lievre M, Thomas C, Hinkal G, Ansieau S, Puisieux A (2008) Generation of breast cancer stem cells through epithelial‐mesenchymal transition. PLoS One 3: e2888 18682804 10.1371/journal.pone.0002888 PMC2492808

[embj2021108647-bib-0160] Moreno‐Bueno G, Portillo F, Cano A (2008) Transcriptional regulation of cell polarity in EMT and cancer. Oncogene 27: 6958–6969 19029937 10.1038/onc.2008.346

[embj2021108647-bib-0161] Moroishi T, Hansen CG, Guan KL (2015) The emerging roles of YAP and TAZ in cancer. Nat Rev Cancer 15: 73–79 25592648 10.1038/nrc3876 PMC4562315

[embj2021108647-bib-0162] Mosa MH, Michels BE, Menche C, Nicolas AM, Darvishi T, Greten FR, Farin HF (2020) A Wnt‐induced phenotypic switch in cancer‐associated fibroblasts inhibits EMT in colorectal cancer. Cancer Res 80: 5569–5582 33055221 10.1158/0008-5472.CAN-20-0263

[embj2021108647-bib-0163] Nath A, Li I, Roberts LR, Chan C (2015) Elevated free fatty acid uptake via CD36 promotes epithelial‐mesenchymal transition in hepatocellular carcinoma. Sci Rep 5: 14752 26424075 10.1038/srep14752 PMC4589791

[embj2021108647-bib-0164] Natsuizaka M, Whelan KA, Kagawa S, Tanaka K, Giroux V, Chandramouleeswaran PM, Long A, Sahu V, Darling DS, Que J*et al* (2017) Interplay between Notch1 and Notch3 promotes EMT and tumor initiation in squamous cell carcinoma. Nat Commun 8: 1758 29170450 10.1038/s41467-017-01500-9 PMC5700926

[embj2021108647-bib-0165] Niessen K, Fu Y, Chang L, Hoodless PA, McFadden D, Karsan A (2008) Slug is a direct Notch target required for initiation of cardiac cushion cellularization. J Cell Biol 182: 315–325 18663143 10.1083/jcb.200710067 PMC2483533

[embj2021108647-bib-0166] Nieto MA, Huang RY, Jackson RA, Thiery JP (2016) Emt: 2016. Cell 166: 21–45 27368099 10.1016/j.cell.2016.06.028

[embj2021108647-bib-0167] Noman MZ, Janji B, Abdou A, Hasmim M, Terry S, Tan TZ, Mami‐Chouaib F, Thiery JP, Chouaib S (2017) The immune checkpoint ligand PD‐L1 is upregulated in EMT‐activated human breast cancer cells by a mechanism involving ZEB‐1 and miR‐200. Oncoimmunology 6: e1263412 28197390 10.1080/2162402X.2016.1263412 PMC5283623

[embj2021108647-bib-0168] Norz D, Grottke A, Bach J, Herzberger C, Hofmann BT, Nashan B, Jucker M, Ewald F (2015) Discontinuing MEK inhibitors in tumor cells with an acquired resistance increases migration and invasion. Cell Signal 27: 2191–2200 26210887 10.1016/j.cellsig.2015.07.016

[embj2021108647-bib-0169] Noy R, Pollard JW (2014) Tumor‐associated macrophages: from mechanisms to therapy. Immunity 41: 49–61 25035953 10.1016/j.immuni.2014.06.010 PMC4137410

[embj2021108647-bib-0170] Ocana OH, Corcoles R, Fabra A, Moreno‐Bueno G, Acloque H, Vega S, Barrallo‐Gimeno A, Cano A, Nieto MA (2012) Metastatic colonization requires the repression of the epithelial‐mesenchymal transition inducer Prrx1. Cancer Cell 22: 709–724 23201163 10.1016/j.ccr.2012.10.012

[embj2021108647-bib-0171] Omabe K, Uduituma S, Igwe D, Omabe M (2021) Deeper insight in metastatic cancer progression;Epithelial‐to‐mesenchymal transition and genomic instability: implications on treatment resistance. Curr Mol Med 21. 10.2174/1566524021666210202114844 33530906

[embj2021108647-bib-0172] Ouzounova M, Lee E, Piranlioglu R, El Andaloussi A, Kolhe R, Demirci MF, Marasco D, Asm I, Chadli A, Hassan KA*et al* (2017) Monocytic and granulocytic myeloid derived suppressor cells differentially regulate spatiotemporal tumour plasticity during metastatic cascade. Nat Commun 8: 14979 28382931 10.1038/ncomms14979 PMC5384228

[embj2021108647-bib-0173] Pascual G, Avgustinova A, Mejetta S, Martín M, Castellanos A, Attolini C‐O, Berenguer A, Prats N, Toll A, Hueto JA*et al* (2017) Targeting metastasis‐initiating cells through the fatty acid receptor CD36. Nature 541: 41–45 27974793 10.1038/nature20791

[embj2021108647-bib-0174] Pastushenko I, Blanpain C (2019) EMT transition states during tumor progression and metastasis. Trends Cell Biol 29: 212–226 30594349 10.1016/j.tcb.2018.12.001

[embj2021108647-bib-0175] Pastushenko I, Brisebarre A, Sifrim A, Fioramonti M, Revenco T, Boumahdi S, Van Keymeulen A, Brown D, Moers V, Lemaire S*et al* (2018) Identification of the tumour transition states occurring during EMT. Nature 556: 463–468 29670281 10.1038/s41586-018-0040-3

[embj2021108647-bib-0176] Pastushenko I, Mauri F, Song Y, de Cock F, Meeusen B, Swedlund B, Impens F, Van Haver D, Opitz M, Thery M*et al* (2021) Fat1 deletion promotes hybrid EMT state, tumour stemness and metastasis. Nature 589: 448–455 33328637 10.1038/s41586-020-03046-1 PMC7612440

[embj2021108647-bib-0177] Pecot CV, Rupaimoole R, Yang Da, Akbani R, Ivan C, Lu C, Wu S, Han H‐D, Shah MY, Rodriguez‐Aguayo C*et al* (2013) Tumour angiogenesis regulation by the miR‐200 family. Nat Commun 4: 2427 24018975 10.1038/ncomms3427 PMC3904438

[embj2021108647-bib-0178] Peter ME (2009) Let‐7 and miR‐200 microRNAs: guardians against pluripotency and cancer progression. Cell Cycle 8: 843–852 19221491 10.4161/cc.8.6.7907 PMC2688687

[embj2021108647-bib-0179] Pistore C, Giannoni E, Colangelo T, Rizzo F, Magnani E, Muccillo L, Giurato G, Mancini M, Rizzo S, Riccardi M*et al* (2017) DNA methylation variations are required for epithelial‐to‐mesenchymal transition induced by cancer‐associated fibroblasts in prostate cancer cells. Oncogene 36: 5551–5566 28581528 10.1038/onc.2017.159

[embj2021108647-bib-0180] Postigo AA (2003) Opposing functions of ZEB proteins in the regulation of the TGFbeta/BMP signaling pathway. EMBO J 22: 2443–2452 12743038 10.1093/emboj/cdg225 PMC155983

[embj2021108647-bib-0181] Prodhomme MK, Pommier RM, Franchet C, Fauvet F, Bergoglio V, Brousset P, Morel AP, Brunac AC, Devouassoux‐Shisheboran M, Petrilli V*et al* (2021) EMT transcription factor ZEB1 represses the mutagenic POLtheta‐mediated end‐joining pathway in breast cancers. Cancer Res 81: 1595–1606 33239429 10.1158/0008-5472.CAN-20-2626

[embj2021108647-bib-0182] Puhr M, Hoefer J, Schafer G, Erb HH, Oh SJ, Klocker H, Heidegger I, Neuwirt H, Culig Z (2012) Epithelial‐to‐mesenchymal transition leads to docetaxel resistance in prostate cancer and is mediated by reduced expression of miR‐200c and miR‐205. Am J Pathol 181: 2188–2201 23041061 10.1016/j.ajpath.2012.08.011

[embj2021108647-bib-0183] Puisieux A, Brabletz T, Caramel J (2014) Oncogenic roles of EMT‐inducing transcription factors. Nat Cell Biol 16: 488–494 24875735 10.1038/ncb2976

[embj2021108647-bib-0184] Puram SV, Tirosh I, Parikh AS, Patel AP, Yizhak K, Gillespie S, Rodman C, Luo CL, Mroz EA, Emerick KS*et al* (2017) Single‐cell transcriptomic analysis of primary and metastatic tumor ecosystems in head and neck cancer. Cell 171: 1611–1624.e24 29198524 10.1016/j.cell.2017.10.044 PMC5878932

[embj2021108647-bib-0185] Ramachandran A, Vizan P, Das D, Chakravarty P, Vogt J, Rogers KW, Muller P, Hinck AP, Sapkota GP, Hill CS (2018) TGF‐beta uses a novel mode of receptor activation to phosphorylate SMAD1/5 and induce epithelial‐to‐mesenchymal transition. Elife 7: e31756 29376829 10.7554/eLife.31756 PMC5832415

[embj2021108647-bib-0186] Rambow F, Rogiers A, Marin‐Bejar O, Aibar S, Femel J, Dewaele M, Karras P, Brown D, Chang YH, Debiec‐Rychter M*et al* (2018) Toward minimal residual disease‐directed therapy in melanoma. Cell 174: 843–855.e19 30017245 10.1016/j.cell.2018.06.025

[embj2021108647-bib-0187] Redfern AD, Spalding LJ, Thompson EW (2018) The Kraken Wakes: induced EMT as a driver of tumour aggression and poor outcome. Clin Exp Metastasis 35: 285–308 29948647 10.1007/s10585-018-9906-x

[embj2021108647-bib-0188] Ren J, Chen Y, Song H, Chen L, Wang R (2013) Inhibition of ZEB1 reverses EMT and chemoresistance in docetaxel‐resistant human lung adenocarcinoma cell line. J Cell Biochem 114: 1395–1403 23255418 10.1002/jcb.24481

[embj2021108647-bib-0189] Reya T, Morrison SJ, Clarke MF, Weissman IL (2001) Stem cells, cancer, and cancer stem cells. Nature 414: 105–111 11689955 10.1038/35102167

[embj2021108647-bib-0190] Richard G, Dalle S, Monet MA, Ligier M, Boespflug A, Pommier RM, de la Fouchardiere A, Perier‐Muzet M, Depaepe L, Barnault R*et al* (2016) ZEB1‐mediated melanoma cell plasticity enhances resistance to MAPK inhibitors. EMBO Mol Med 8: 1143–1161 27596438 10.15252/emmm.201505971 PMC5048365

[embj2021108647-bib-0191] Ridley AJ (2011) Life at the leading edge. Cell 145: 1012–1022 21703446 10.1016/j.cell.2011.06.010

[embj2021108647-bib-0192] Ruh M, Stemmler MP, Frisch I, Fuchs K, van Roey R, Kleemann J, Roas M, Schuhwerk H, Eccles RL, Agaimy A*et al* (2021) The EMT transcription factor ZEB1 blocks osteoblastic differentiation in bone development and osteosarcoma. J Pathol 254: 199–211 33675037 10.1002/path.5659

[embj2021108647-bib-0193] Ruscetti M, Quach B, Dadashian EL, Mulholland DJ, Wu H (2015) Tracking and functional characterization of epithelial‐mesenchymal transition and mesenchymal tumor cells during prostate cancer metastasis. Cancer Res 75: 2749–2759 25948589 10.1158/0008-5472.CAN-14-3476 PMC4490048

[embj2021108647-bib-0194] Sahai E, Astsaturov I, Cukierman E, DeNardo DG, Egeblad M, Evans RM, Fearon D, Greten FR, Hingorani SR, Hunter T*et al* (2020) A framework for advancing our understanding of cancer‐associated fibroblasts. Nat Rev Cancer 20: 174–186 31980749 10.1038/s41568-019-0238-1 PMC7046529

[embj2021108647-bib-0195] Sakata J, Utsumi F, Suzuki S, Niimi K, Yamamoto E, Shibata K, Senga T, Kikkawa F, Kajiyama H (2017) Inhibition of ZEB1 leads to inversion of metastatic characteristics and restoration of paclitaxel sensitivity of chronic chemoresistant ovarian carcinoma cells. Oncotarget 8: 99482–99494 29245917 10.18632/oncotarget.20107 PMC5725108

[embj2021108647-bib-0196] Salazar Y, Zheng X, Brunn D, Raifer H, Picard F, Zhang Y, Winter H, Guenther S, Weigert A, Weigmann B*et al* (2020) Microenvironmental Th9 and Th17 lymphocytes induce metastatic spreading in lung cancer. J Clin Invest 130: 3560–3575 32229721 10.1172/JCI124037 PMC7324180

[embj2021108647-bib-0197] Sanchez‐Tillo E, de Barrios O, Siles L, Cuatrecasas M, Castells A, Postigo A (2011) beta‐catenin/TCF4 complex induces the epithelial‐to‐mesenchymal transition (EMT)‐activator ZEB1 to regulate tumor invasiveness. Proc Natl Acad Sci U S A 108: 19204–19209 22080605 10.1073/pnas.1108977108 PMC3228467

[embj2021108647-bib-0198] Santamaria PG, Moreno‐Bueno G, Cano A (2019) Contribution of epithelial plasticity to therapy resistance. J Clin Med 8: 676 10.3390/jcm8050676 PMC6571660 31091749

[embj2021108647-bib-0199] Santisteban M, Reiman JM, Asiedu MK, Behrens MD, Nassar A, Kalli KR, Haluska P, Ingle JN, Hartmann LC, Manjili MH*et al* (2009) Immune‐induced epithelial to mesenchymal transition in vivo generates breast cancer stem cells. Cancer Res 69: 2887–2895 19276366 10.1158/0008-5472.CAN-08-3343 PMC2664865

[embj2021108647-bib-0200] Saxena M, Stephens MA, Pathak H, Rangarajan A (2011) Transcription factors that mediate epithelial‐mesenchymal transition lead to multidrug resistance by upregulating ABC transporters. Cell Death Dis 2: e179 21734725 10.1038/cddis.2011.61 PMC3199722

[embj2021108647-bib-0201] Scheel C, Eaton E, Li S‐J, Chaffer C, Reinhardt F, Kah K‐J, Bell G, Guo W, Rubin J, Richardson A*et al* (2011) Paracrine and autocrine signals induce and maintain mesenchymal and stem cell states in the breast. Cell 145: 926–940 21663795 10.1016/j.cell.2011.04.029 PMC3930331

[embj2021108647-bib-0202] Sciacovelli M, Frezza C (2017) Fumarate drives EMT in renal cancer. Cell Death Differ 24: 1–2 27886164 10.1038/cdd.2016.137 PMC5260501

[embj2021108647-bib-0203] Sciacovelli M, Gonçalves E, Johnson TI, Zecchini VR, da Costa ASH, Gaude E, Drubbel AV, Theobald SJ, Abbo SR, Tran MGB*et al* (2016) Fumarate is an epigenetic modifier that elicits epithelial‐to‐mesenchymal transition. Nature 537: 544–547 27580029 10.1038/nature19353 PMC5136292

[embj2021108647-bib-0204] Seoane J, Gomis RR (2017) TGF‐beta family signaling in tumor suppression and cancer progression. Cold Spring Harb Perspect Biol 9: a022277 28246180 10.1101/cshperspect.a022277 PMC5710110

[embj2021108647-bib-0205] Sequist LV, Waltman BA, Dias‐Santagata D, Digumarthy S, Turke AB, Fidias P, Bergethon K, Shaw AT, Gettinger S, Cosper AK*et al* (2011) Genotypic and histological evolution of lung cancers acquiring resistance to EGFR inhibitors. Sci Transl Med 3: 75ra26 10.1126/scitranslmed.3002003 PMC3132801 21430269

[embj2021108647-bib-0206] Shaffer SM, Dunagin MC, Torborg SR, Torre EA, Emert B, Krepler C, Beqiri M, Sproesser K, Brafford PA, Xiao M*et al* (2017) Rare cell variability and drug‐induced reprogramming as a mode of cancer drug resistance. Nature 546: 431–435 28607484 10.1038/nature22794 PMC5542814

[embj2021108647-bib-0207] Shao D, Xue W, Krall E, Bhutkar A, Piccioni F, Wang X, Schinzel A, Sood S, Rosenbluh J, Kim J*et al* (2014) KRAS and YAP1 converge to regulate EMT and tumor survival. Cell 158: 171–184 24954536 10.1016/j.cell.2014.06.004 PMC4110062

[embj2021108647-bib-0208] Shaul Y, Freinkman E, Comb W, Cantor J, Tam W, Thiru P, Kim D, Kanarek N, Pacold M, Chen W*et al* (2014) Dihydropyrimidine accumulation is required for the epithelial‐mesenchymal transition. Cell 158: 1094–1109 25171410 10.1016/j.cell.2014.07.032 PMC4250222

[embj2021108647-bib-0209] Sheng W, Shi X, Lin Y, Tang J, Jia C, Cao R, Sun J, Wang G, Zhou L, Dong M (2020) Musashi2 promotes EGF‐induced EMT in pancreatic cancer via ZEB1‐ERK/MAPK signaling. J Exp Clin Cancer Res 39: 16 31952541 10.1186/s13046-020-1521-4 PMC6967093

[embj2021108647-bib-0210] Shibue T, Brooks MW, Weinberg RA (2013) An integrin‐linked machinery of cytoskeletal regulation that enables experimental tumor initiation and metastatic colonization. Cancer Cell 24: 481–498 24035453 10.1016/j.ccr.2013.08.012 PMC3864118

[embj2021108647-bib-0211] Shibue T, Weinberg RA (2009) Integrin beta1‐focal adhesion kinase signaling directs the proliferation of metastatic cancer cells disseminated in the lungs. Proc Natl Acad Sci U S A 106: 10290–10295 19502425 10.1073/pnas.0904227106 PMC2700942

[embj2021108647-bib-0212] Shibue T, Weinberg RA (2017) EMT, CSCs, and drug resistance: the mechanistic link and clinical implications. Nat Rev Clin Oncol 14: 611–629 28397828 10.1038/nrclinonc.2017.44 PMC5720366

[embj2021108647-bib-0213] Shimono Y, Zabala M, Cho RW, Lobo N, Dalerba P, Qian D, Diehn M, Liu H, Panula SP, Chiao E*et al* (2009) Downregulation of miRNA‐200c links breast cancer stem cells with normal stem cells. Cell 138: 592–603 19665978 10.1016/j.cell.2009.07.011 PMC2731699

[embj2021108647-bib-0214] Shin S, Dimitri CA, Yoon SO, Dowdle W, Blenis J (2010) ERK2 but not ERK1 induces epithelial‐to‐mesenchymal transformation via DEF motif‐dependent signaling events. Mol Cell 38: 114–127 20385094 10.1016/j.molcel.2010.02.020 PMC2854677

[embj2021108647-bib-0215] Shintani Y, Fujiwara A, Kimura T, Kawamura T, Funaki S, Minami M, Okumura M (2016) IL‐6 secreted from cancer‐associated fibroblasts mediates chemoresistance in NSCLC by increasing epithelial‐mesenchymal transition signaling. J Thorac Oncol 11: 1482–1492 27287412 10.1016/j.jtho.2016.05.025

[embj2021108647-bib-0216] Shirakihara T, Saitoh M, Miyazono K (2007) Differential regulation of epithelial and mesenchymal markers by deltaEF1 proteins in epithelial mesenchymal transition induced by TGF‐beta. Mol Biol Cell 18: 3533–3544 17615296 10.1091/mbc.E07-03-0249 PMC1951739

[embj2021108647-bib-0217] Siemens H, Jackstadt R, Hunten S, Kaller M, Menssen A, Gotz U, Hermeking H (2011) miR‐34 and SNAIL form a double‐negative feedback loop to regulate epithelial‐mesenchymal transitions. Cell Cycle 10: 4256–4271 22134354 10.4161/cc.10.24.18552

[embj2021108647-bib-0218] Singh A, Greninger P, Rhodes D, Koopman L, Violette S, Bardeesy N, Settleman J (2009) A gene expression signature associated with "K‐Ras addiction" reveals regulators of EMT and tumor cell survival. Cancer Cell 15: 489–500 19477428 10.1016/j.ccr.2009.03.022 PMC2743093

[embj2021108647-bib-0219] Singh A, Settleman J (2010) EMT, cancer stem cells and drug resistance: an emerging axis of evil in the war on cancer. Oncogene 29: 4741–4751 20531305 10.1038/onc.2010.215 PMC3176718

[embj2021108647-bib-0220] Slowicka K, Petta I, Blancke G, Hoste E, Dumas E, Sze M, Vikkula H, Radaelli E, Haigh JJ, Jonckheere S*et al* (2020) Zeb2 drives invasive and microbiota‐dependent colon carcinoma. Nature Cancer 1: 620–634 10.1038/s43018-020-0070-2 35121975

[embj2021108647-bib-0221] Song NA, Jing W, Li CE, Bai M, Cheng YU, Li H, Hou K, Li Y, Wang K, Li Z*et al* (2018) ZEB1 inhibition sensitizes cells to the ATR inhibitor VE‐821 by abrogating epithelial‐mesenchymal transition and enhancing DNA damage. Cell Cycle 17: 595–604 29157079 10.1080/15384101.2017.1404206 PMC5969561

[embj2021108647-bib-0222] Spaderna S, Schmalhofer O, Hlubek F, Berx G, Eger A, Merkel S, Jung A, Kirchner T, Brabletz T (2006) A transient, EMT‐linked loss of basement membranes indicates metastasis and poor survival in colorectal cancer. Gastroenterology 131: 830–840 16952552 10.1053/j.gastro.2006.06.016

[embj2021108647-bib-0223] Spaderna S, Schmalhofer O, Wahlbuhl M, Dimmler A, Bauer K, Sultan A, Hlubek F, Jung A, Strand D, Eger A*et al* (2008) The transcriptional repressor ZEB1 promotes metastasis and loss of cell polarity in cancer. Cancer Res 68: 537–544 18199550 10.1158/0008-5472.CAN-07-5682

[embj2021108647-bib-0224] Stemmler MP, Eccles RL, Brabletz S, Brabletz T (2019) Non‐redundant functions of EMT transcription factors. Nat Cell Biol 21: 102–112 30602760 10.1038/s41556-018-0196-y

[embj2021108647-bib-0225] Su J, Zhang A, Shi Z, Ma F, Pu P, Wang T, Zhang J, Kang C, Zhang Q (2012) MicroRNA‐200a suppresses the Wnt/beta‐catenin signaling pathway by interacting with beta‐catenin. Int J Oncol 40: 1162–1170 22211245 10.3892/ijo.2011.1322 PMC3584589

[embj2021108647-bib-0226] Sullivan NJ, Sasser AK, Axel AE, Vesuna F, Raman V, Ramirez N, Oberyszyn TM, Hall BM (2009) Interleukin‐6 induces an epithelial‐mesenchymal transition phenotype in human breast cancer cells. Oncogene 28: 2940–2947 19581928 10.1038/onc.2009.180 PMC5576031

[embj2021108647-bib-0227] Sun D, Luo T, Dong P, Zhang N, Chen J, Zhang S, Dong L, Janssen HLA, Zhang S (2020) M2‐polarized tumor‐associated macrophages promote epithelial‐mesenchymal transition via activation of the AKT3/PRAS40 signaling pathway in intrahepatic cholangiocarcinoma. J Cell Biochem 121: 2828–2838 31692069 10.1002/jcb.29514

[embj2021108647-bib-0228] Sun NY, Yang MH (2020) Metabolic reprogramming and epithelial‐mesenchymal plasticity: opportunities and challenges for cancer therapy. Front Oncol 10: 792 32509584 10.3389/fonc.2020.00792 PMC7252305

[embj2021108647-bib-0229] Sundararajan V, Gengenbacher N, Stemmler MP, Kleemann JA, Brabletz T, Brabletz S (2015) The ZEB1/miR‐200c feedback loop regulates invasion via actin interacting proteins MYLK and TKS5. Oncotarget 6: 27083–27096 26334100 10.18632/oncotarget.4807 PMC4694975

[embj2021108647-bib-0230] Takano S, Reichert M, Bakir B, Das KK, Nishida T, Miyazaki M, Heeg S, Collins MA, Marchand B, Hicks PD*et al* (2016) Prrx1 isoform switching regulates pancreatic cancer invasion and metastatic colonization. Genes Dev 30: 233–247 26773005 10.1101/gad.263327.115 PMC4719312

[embj2021108647-bib-0231] Taki M, Abiko K, Baba T, Hamanishi J, Yamaguchi K, Murakami R, Yamanoi K, Horikawa N, Hosoe Y, Nakamura E*et al* (2018) Snail promotes ovarian cancer progression by recruiting myeloid‐derived suppressor cells via CXCR2 ligand upregulation. Nat Commun 9: 1685 29703902 10.1038/s41467-018-03966-7 PMC5923228

[embj2021108647-bib-0232] Tauriello DVF, Palomo‐Ponce S, Stork D, Berenguer‐Llergo A, Badia‐Ramentol J, Iglesias M, Sevillano M, Ibiza S, Canellas A, Hernando‐Momblona X*et al* (2018) TGFbeta drives immune evasion in genetically reconstituted colon cancer metastasis. Nature 554: 538–543 29443964 10.1038/nature25492

[embj2021108647-bib-0233] Terry S, Savagner P, Ortiz‐Cuaran S, Mahjoubi L, Saintigny P, Thiery JP, Chouaib S (2017) New insights into the role of EMT in tumor immune escape. Mol Oncol 11: 824–846 28614624 10.1002/1878-0261.12093 PMC5496499

[embj2021108647-bib-0234] Thiery JP, Acloque H, Huang RY, Nieto MA (2009) Epithelial‐mesenchymal transitions in development and disease. Cell 139: 871–890 19945376 10.1016/j.cell.2009.11.007

[embj2021108647-bib-0235] Tian YC, Chen YC, Chang CT, Hung CC, Wu MS, Phillips A, Yang CW (2007) Epidermal growth factor and transforming growth factor‐beta1 enhance HK‐2 cell migration through a synergistic increase of matrix metalloproteinase and sustained activation of ERK signaling pathway. Exp Cell Res 313: 2367–2377 17467690 10.1016/j.yexcr.2007.03.022

[embj2021108647-bib-0236] Timmerman LA, Grego‐Bessa J, Raya A, Bertran E, Perez‐Pomares JM, Diez J, Aranda S, Palomo S, McCormick F, Izpisua‐Belmonte JC*et al* (2004) Notch promotes epithelial‐mesenchymal transition during cardiac development and oncogenic transformation. Genes Dev 18: 99–115 14701881 10.1101/gad.276304 PMC314285

[embj2021108647-bib-0237] Tripathi SC, Peters HL, Taguchi A, Katayama H, Wang H, Momin A, Jolly MK, Celiktas M, Rodriguez‐Canales J, Liu H*et al* (2016) Immunoproteasome deficiency is a feature of non‐small cell lung cancer with a mesenchymal phenotype and is associated with a poor outcome. Proc Natl Acad Sci U S A 113: E1555–E1564 26929325 10.1073/pnas.1521812113 PMC4801290

[embj2021108647-bib-0238] Trovato R, Cane S, Petrova V, Sartoris S, Ugel S, De Sanctis F (2020) The engagement between MDSCs and metastases: partners in crime. Front Oncol 10: 165 32133298 10.3389/fonc.2020.00165 PMC7040035

[embj2021108647-bib-0239] Tsai JH, Donaher JL, Murphy DA, Chau S, Yang J (2012) Spatiotemporal regulation of epithelial‐mesenchymal transition is essential for squamous cell carcinoma metastasis. Cancer Cell 22: 725–736 23201165 10.1016/j.ccr.2012.09.022 PMC3522773

[embj2021108647-bib-0240] Tulchinsky E, Demidov O, Kriajevska M, Barlev NA, Imyanitov E (2019) EMT: a mechanism for escape from EGFR‐targeted therapy in lung cancer. Biochim Biophys Acta Rev Cancer 1871: 29–39 30419315 10.1016/j.bbcan.2018.10.003

[embj2021108647-bib-0241] Uttamsingh S, Bao X, Nguyen KT, Bhanot M, Gong J, Chan JL, Liu F, Chu TT, Wang LH (2008) Synergistic effect between EGF and TGF‐beta1 in inducing oncogenic properties of intestinal epithelial cells. Oncogene 27: 2626–2634 17982486 10.1038/sj.onc.1210915

[embj2021108647-bib-0242] Vega S, Morales AV, Ocana OH, Valdes F, Fabregat I, Nieto MA (2004) Snail blocks the cell cycle and confers resistance to cell death. Genes Dev 18: 1131–1143 15155580 10.1101/gad.294104 PMC415638

[embj2021108647-bib-0243] Vincent T, Neve EP, Johnson JR, Kukalev A, Rojo F, Albanell J, Pietras K, Virtanen I, Philipson L, Leopold PL*et al* (2009) A SNAIL1‐SMAD3/4 transcriptional repressor complex promotes TGF‐beta mediated epithelial‐mesenchymal transition. Nat Cell Biol 11: 943–950 19597490 10.1038/ncb1905 PMC3769970

[embj2021108647-bib-0244] Viswanathan VS, Ryan MJ, Dhruv HD, Gill S, Eichhoff OM, Seashore‐Ludlow B, Kaffenberger SD, Eaton JK, Shimada K, Aguirre AJ*et al* (2017) Dependency of a therapy‐resistant state of cancer cells on a lipid peroxidase pathway. Nature 547: 453–457 28678785 10.1038/nature23007 PMC5667900

[embj2021108647-bib-0245] Wang H, Xiang D, Liu B, He A, Randle HJ, Zhang KX, Dongre A, Sachs N, Clark AP, Tao L*et al* (2019a) Inadequate DNA damage repair promotes mammary transdifferentiation, leading to BRCA1 breast cancer. Cell 178: 135–151.e19 31251913 10.1016/j.cell.2019.06.002 PMC6716369

[embj2021108647-bib-0246] Wang Y, Chen J, Yang L, Li J, Wu W, Huang M, Lin L, Su S (2019b) Tumor‐contacted neutrophils promote metastasis by a CD90‐TIMP‐1 JUXTACRINE‐paracrine loop. Clin Cancer Res 25: 1957–1969 30482778 10.1158/1078-0432.CCR-18-2544

[embj2021108647-bib-0247] Wang Y, Liao R, Chen X, Ying X, Chen G, Li M, Dong C (2020) Twist‐mediated PAR1 induction is required for breast cancer progression and metastasis by inhibiting Hippo pathway. Cell Death Dis 11: 520 32647142 10.1038/s41419-020-2725-4 PMC7347637

[embj2021108647-bib-0248] Wellner U, Schubert J, Burk UC, Schmalhofer O, Zhu F, Sonntag A, Waldvogel B, Vannier C, Darling D, Hausen AZ*et al* (2009) The EMT‐activator ZEB1 promotes tumorigenicity by repressing stemness‐inhibiting microRNAs. Nat Cell Biol 11: 1487–1495 19935649 10.1038/ncb1998

[embj2021108647-bib-0249] Wilson MM, Weinberg RA, Lees JA, Guen VJ (2020) Emerging mechanisms by which EMT programs control stemness. Trends Cancer 6: 775–780 32312682 10.1016/j.trecan.2020.03.011

[embj2021108647-bib-0250] Wu DW, Lee MC, Hsu NY, Wu TC, Wu JY, Wang YC, Cheng YW, Chen CY, Lee H (2015) FHIT loss confers cisplatin resistance in lung cancer via the AKT/NF‐kappaB/Slug‐mediated PUMA reduction. Oncogene 34: 2505–2515 24998847 10.1038/onc.2014.184

[embj2021108647-bib-0251] Wu L, Zhang XH (2020) Tumor‐associated neutrophils and macrophages‐heterogenous but not chaotic. Front Immunol 11: 553967 33343560 10.3389/fimmu.2020.553967 PMC7738476

[embj2021108647-bib-0252] Xie M, Zhang L, He CS, Xu F, Liu JL, Hu ZH, Zhao LP, Tian Y (2012) Activation of Notch‐1 enhances epithelial‐mesenchymal transition in gefitinib‐acquired resistant lung cancer cells. J Cell Biochem 113: 1501–1513 22173954 10.1002/jcb.24019

[embj2021108647-bib-0253] Xu Y, Lee DK, Feng Z, Xu Y, Bu W, Li Y, Liao L, Xu J (2017) Breast tumor cell‐specific knockout of Twist1 inhibits cancer cell plasticity, dissemination, and lung metastasis in mice. Proc Natl Acad Sci U S A 114: 11494–11499 29073077 10.1073/pnas.1618091114 PMC5664488

[embj2021108647-bib-0254] Yang J, Antin P, Berx G, Blanpain C, Brabletz T, Bronner M, Campbell K, Cano A, Casanova J, Christofori G*et al* (2020) Guidelines and definitions for research on epithelial‐mesenchymal transition. Nat Rev Mol Cell Biol 21: 341–352 32300252 10.1038/s41580-020-0237-9 PMC7250738

[embj2021108647-bib-0255] Yang J, Mani SA, Donaher JL, Ramaswamy S, Itzykson RA, Come C, Savagner P, Gitelman I, Richardson A, Weinberg RA (2004) Twist, a master regulator of morphogenesis, plays an essential role in tumor metastasis. Cell 117: 927–939 15210113 10.1016/j.cell.2004.06.006

[embj2021108647-bib-0256] Yang L, Lin C, Liu ZR (2006) P68 RNA helicase mediates PDGF‐induced epithelial mesenchymal transition by displacing Axin from beta‐catenin. Cell 127: 139–155 17018282 10.1016/j.cell.2006.08.036

[embj2021108647-bib-0257] Yang M‐H, Hsu D‐S, Wang H‐W, Wang H‐J, Lan H‐Y, Yang W‐H, Huang C‐H, Kao S‐Y, Tzeng C‐H, Tai S‐K*et al* (2010) Bmi1 is essential in Twist1‐induced epithelial‐mesenchymal transition. Nat Cell Biol 12: 982–992 20818389 10.1038/ncb2099

[embj2021108647-bib-0258] Yang MH, Wu MZ, Chiou SH, Chen PM, Chang SY, Liu CJ, Teng SC, Wu KJ (2008) Direct regulation of TWIST by HIF‐1alpha promotes metastasis. Nat Cell Biol 10: 295–305 18297062 10.1038/ncb1691

[embj2021108647-bib-0259] Ye X, Tam WL, Shibue T, Kaygusuz Y, Reinhardt F, Ng Eaton E, Weinberg RA (2015) Distinct EMT programs control normal mammary stem cells and tumour‐initiating cells. Nature 525: 256–260 26331542 10.1038/nature14897 PMC4764075

[embj2021108647-bib-0260] Yilmaz M, Christofori G (2009) EMT, the cytoskeleton, and cancer cell invasion. Cancer Metastasis Rev 28: 15–33 19169796 10.1007/s10555-008-9169-0

[embj2021108647-bib-0261] Yochum ZA, Cades J, Wang H, Chatterjee S, Simons BW, O’Brien JP, Khetarpal SK, Lemtiri‐Chlieh G, Myers KV, Huang E‐B*et al* (2019) Targeting the EMT transcription factor TWIST1 overcomes resistance to EGFR inhibitors in EGFR‐mutant non‐small‐cell lung cancer. Oncogene 38: 656–670 30171258 10.1038/s41388-018-0482-y PMC6358506

[embj2021108647-bib-0262] Youssef KK, Nieto MA (2020) Glucose metabolism takes center stage in epithelial‐mesenchymal plasticity. Dev Cell 53: 133–135 32315608 10.1016/j.devcel.2020.03.021

[embj2021108647-bib-0263] Yu M, Bardia A, Wittner BS, Stott SL, Smas ME, Ting DT, Isakoff SJ, Ciciliano JC, Wells MN, Shah AM*et al* (2013) Circulating breast tumor cells exhibit dynamic changes in epithelial and mesenchymal composition. Science 339: 580–584 23372014 10.1126/science.1228522 PMC3760262

[embj2021108647-bib-0264] Yu Y, Xiao CH, Tan LD, Wang QS, Li XQ, Feng YM (2014) Cancer‐associated fibroblasts induce epithelial‐mesenchymal transition of breast cancer cells through paracrine TGF‐beta signalling. Br J Cancer 110: 724–732 24335925 10.1038/bjc.2013.768 PMC3915130

[embj2021108647-bib-0265] Yuan X, Wu H, Han N, Xu H, Chu Q, Yu S, Chen Y, Wu K (2014) Notch signaling and EMT in non‐small cell lung cancer: biological significance and therapeutic application. J Hematol Oncol 7: 87 25477004 10.1186/s13045-014-0087-z PMC4267749

[embj2021108647-bib-0266] Zanconato F, Forcato M, Battilana G, Azzolin L, Quaranta E, Bodega B, Rosato A, Bicciato S, Cordenonsi M, Piccolo S (2015) Genome‐wide association between YAP/TAZ/TEAD and AP‐1 at enhancers drives oncogenic growth. Nat Cell Biol 17: 1218–1227 26258633 10.1038/ncb3216 PMC6186417

[embj2021108647-bib-0267] Zavadil J, Bottinger EP (2005) TGF‐beta and epithelial‐to‐mesenchymal transitions. Oncogene 24: 5764–5774 16123809 10.1038/sj.onc.1208927

[embj2021108647-bib-0268] Zhang D, Yang L, Liu X, Gao J, Liu T, Yan Q, Yang X (2020) Hypoxia modulates stem cell properties and induces EMT through N‐glycosylation of EpCAM in breast cancer cells. J Cell Physiol 235: 3626–3633 31584203 10.1002/jcp.29252 PMC13482652

[embj2021108647-bib-0269] Zhang H, Liu CY, Zha ZY, Zhao B, Yao J, Zhao S, Xiong Y, Lei QY, Guan KL (2009) TEAD transcription factors mediate the function of TAZ in cell growth and epithelial‐mesenchymal transition. J Biol Chem 284: 13355–13362 19324877 10.1074/jbc.M900843200 PMC2679435

[embj2021108647-bib-0270] Zhang L, Liao Y, Tang L (2019a) MicroRNA‐34 family: a potential tumor suppressor and therapeutic candidate in cancer. J Exp Clin Cancer Res 38: 53 30717802 10.1186/s13046-019-1059-5 PMC6360685

[embj2021108647-bib-0271] Zhang P, Wei Y, Wang Li, Debeb BG, Yuan Y, Zhang J, Yuan J, Wang M, Chen D, Sun Y*et al* (2014) ATM‐mediated stabilization of ZEB1 promotes DNA damage response and radioresistance through CHK1. Nat Cell Biol 16: 864–875 25086746 10.1038/ncb3013 PMC4150825

[embj2021108647-bib-0272] Zhang T, Liu L, Lai W, Zeng Y, Xu H, Lan Q, Su P, Chu Z (2019b) Interaction with tumorassociated macrophages promotes PRL3induced invasion of colorectal cancer cells via MAPK pathwayinduced EMT and NFkappaB signalinginduced angiogenesis. Oncol Rep 41: 2790–2802 30864736 10.3892/or.2019.7049 PMC6448091

[embj2021108647-bib-0273] Zhang X, Zhang Z, Zhang Q, Zhang Q, Sun P, Xiang R, Ren G, Yang S (2018) ZEB1 confers chemotherapeutic resistance to breast cancer by activating ATM. Cell Death Dis 9: 57 29352223 10.1038/s41419-017-0087-3 PMC5833408

[embj2021108647-bib-0274] Zhang Z, Lee JC, Lin L, Olivas V, Au V, LaFramboise T, Abdel‐Rahman M, Wang X, Levine AD, Rho JK*et al* (2012) Activation of the AXL kinase causes resistance to EGFR‐targeted therapy in lung cancer. Nat Genet 44: 852–860 22751098 10.1038/ng.2330 PMC3408577

[embj2021108647-bib-0275] Zhao L, Ji G, Le X, Luo Z, Wang C, Feng M, Xu L, Zhang Y, Lau Wb, Lau B*et al* (2017) An integrated analysis identifies STAT4 as a key regulator of ovarian cancer metastasis. Oncogene 36: 3384–3396 28114283 10.1038/onc.2016.487

[embj2021108647-bib-0276] Zheng X, Carstens JL, Kim J, Scheible M, Kaye J, Sugimoto H, Wu CC, LeBleu VS, Kalluri R (2015) Epithelial‐to‐mesenchymal transition is dispensable for metastasis but induces chemoresistance in pancreatic cancer. Nature 527: 525–530 26560028 10.1038/nature16064 PMC4849281

[embj2021108647-bib-0277] Zheng X, Lu G, Yao Y, Gu W (2019) An autocrine IL‐6/IGF‐1R loop mediates EMT and promotes tumor growth in non‐small cell lung cancer. Int J Biol Sci 15: 1882–1891 31523190 10.7150/ijbs.31999 PMC6743301

[embj2021108647-bib-0278] Zhou BP, Deng J, Xia W, Xu J, Li YM, Gunduz M, Hung MC (2004) Dual regulation of Snail by GSK‐3beta‐mediated phosphorylation in control of epithelial‐mesenchymal transition. Nat Cell Biol 6: 931–940 15448698 10.1038/ncb1173

[embj2021108647-bib-0279] Zhu G, Li X, Guo B, Ke Q, Dong M, Li F (2016) PAK5‐mediated E47 phosphorylation promotes epithelial‐mesenchymal transition and metastasis of colon cancer. Oncogene 35: 1943–1954 26212009 10.1038/onc.2015.259

[embj2021108647-bib-0280] Zhu H, Gu Y, Xue Y, Yuan M, Cao X, Liu Q (2017) CXCR2(+) MDSCs promote breast cancer progression by inducing EMT and activated T cell exhaustion. Oncotarget 8: 114554–114567 29383101 10.18632/oncotarget.23020 PMC5777713

[embj2021108647-bib-0281] Zou W, Wolchok JD, Chen L (2016) PD‐L1 (B7–H1) and PD‐1 pathway blockade for cancer therapy: Mechanisms, response biomarkers, and combinations. Sci Transl Med 8: 328rv324 10.1126/scitranslmed.aad7118 PMC4859220 26936508

